# Supplementary material for: Psychological distress and health-related quality of life in patients after hospitalization during the COVID-19 pandemic: A single-center, observational study
Source: PLoS One. 2021 Aug 11;16(8):e0255774. doi: 10.1371/journal.pone.0255774 (PMC8357130; doi:10.1371/journal.pone.0255774)
Supplement: S1 Table — (DOCX) [file pone.0255774.s001.docx]

| S1 Table. Possible predictors for psychological distress. | |
| --- | --- |
| **Factor** | **Evidence in literature** |
| Age | Younger age is associated with higher risk for psychological distress (anxiety, depression and PTSD). (1-3)  Elderly patients have a lower stress response, but are more prone to depression and loneliness. (4, 5) |
| Gender | Females might be more prone to anxiety and depression and PTSD. (1, 2, 6, 7) |
| Educational level | Lower educational level is associated with a higher risk for psychological distress. (3) |
| Ethnicity | People with a non-western background are more prone to develop PTSD and are more prone to anxiety disorders. (2, 3) |
| Duration of admission | Longer duration of admission is associated with more risk for psychological distress. (1, 2, 8) |
| ICU admission yes/no | ICU admission is associated with a higher risk for psychological distress. (1, 6, 7, 9) |
| Severity of disease | Critically ill patients are more prone to develop psychological disorders. (1, 2, 8) |
| COVID diagnosis yes/no | COVID-19 might be associated with a higher risk for psychological distress based on previous results in SARS/MERS population and early results 1 month after discharge among COVID-19 patients.  (4, 10-12) |
| Work before admission yes/no | Retirement is associated with a lower risk for PTSD. (4)  Unemployment and retirement is associated with a higher risk of psychological distress. (3) |

1. Rabiee A, Nikayin S, Hashem MD, et al. Depressive Symptoms After Critical Illness: A Systematic Review and Meta-Analysis. Crit Care Med 2016;44(9):1744-1753.

2. Visser E, Gosens T, Den Oudsten BL, et al. The course, prediction, and treatment of acute and posttraumatic stress in trauma patients: a systematic review. Journal of Trauma and Acute Care Surgery 2017;82(6):1158-1183.

3. De Graaf R, Ten Have M, van Dorsselaer S. De psychische gezondheid van de Nederlandse bevolking. Nemesis-2: Opzet en eerste resultaten, Trimbos-Instituut, Utrecht 2010.

4. Cai X, Hu X, Ekumi IO, et al. Psychological distress and its correlates among COVID-19 survivors during early convalescence across age groups. The American Journal of Geriatric Psychiatry 2020;28(10):1030-1039.

5. Zhang J, Yang Z, Wang X, et al. The relationship between resilience, anxiety and depression among patients with mild symptoms of COVID‐19 in China: A cross‐sectional study. J Clin Nurs 2020;29(21-22):4020-4029.

6. Buckland SA, Pozehl B, Yates B. Depressive symptoms in women with Coronary Heart Disease: A systematic review of the longitudinal literature. J Cardiovasc Nurs 2019;34(1):52-59.

7. de Vries GJ, Olff M. The lifetime prevalence of traumatic events and posttraumatic stress disorder in the Netherlands. Journal of Traumatic Stress: Official Publication of The International Society for Traumatic Stress Studies 2009;22(4):259-267.

8. Davydow DS, Desai SV, Needham DM, et al. Psychiatric morbidity in survivors of the acute respiratory distress syndrome: a systematic review. Psychosom Med 2008;70(4):512-519.

9. Nikayin S, Rabiee A, Hashem MD, et al. Anxiety symptoms in survivors of critical illness: a systematic review and meta-analysis. Gen Hosp Psychiatry 2016;43:23-29.

10. Rogers JP, Chesney E, Oliver D, et al. Psychiatric and neuropsychiatric presentations associated with severe coronavirus infections: a systematic review and meta-analysis with comparison to the COVID-19 pandemic. Lancet Psychiatry 2020;7(7):611-627.

11. Mazza MG, De Lorenzo R, Conte C, et al. Anxiety and depression in COVID-19 survivors: Role of inflammatory and clinical predictors. Brain Behav Immun 2020;89:594-600.

12. Krishnamoorthy Y, Nagarajan R, Saya GK, et al. Prevalence of psychological morbidities among general population, healthcare workers and COVID-19 patients amidst the COVID-19 pandemic: A systematic review and meta-analysis. Psychiatry Res 2020;293:113382.
