# Supplementary material for: Psychological distress and health-related quality of life in patients after hospitalization during the COVID-19 pandemic: A single-center, observational study
Source: PLoS One. 2021 Aug 11;16(8):e0255774. doi: 10.1371/journal.pone.0255774 (PMC8357130; doi:10.1371/journal.pone.0255774)
Supplement: S2 Table — (DOCX) [file pone.0255774.s002.docx]

| **S2 Table.** Reliability of the impact of event scale-revised, the hospital anxiety and depression scale, the short-form 36, and the EQ-5D in the entire population and stratifications. | | | | | | | | | |
| --- | --- | --- | --- | --- | --- | --- | --- | --- | --- |
|  |  |  |  | **Total cohort** | |  | **COVID-19 cohort** | |  |
| **Questionnaires** |  | Total cohort |  | COVID-19 | non-COVID-19 |  | ICU | non-ICU |  |
| Impact of Event Scale – Revised (IES-R) |  | 0.937 |  | 0.939 | 0.922 |  | 0.912 | 0.945 |  |
| Hospital Anxiety and Depression Scale (HADS) |  | 0.935 |  | 0.934 | 0.933 |  | 0.923 | 0.937 |  |
| Short-Form 36 (SF-36) |  | 0.949 |  | 0.949 | 0.948 |  | 0.947 | 0.950 |  |
| European Quality of Life – 5 dimensions (EQ-5D-5L) |  | 0.834 |  | 0.822 | 0.837 |  | 0.825 | 0.821 |  |
| Values represent the Cronbach’s Alphas, and give an indication of the internal reliability of the questionnaire. A Cronbach’s alpha ≥ 0.9 is considered excellent, a Cronbach’s alpha ≥ 0.8 is considered good. | | | | | | | | | |
