# Supplementary material for: Psychological distress and health-related quality of life in patients after hospitalization during the COVID-19 pandemic: A single-center, observational study
Source: PLoS One. 2021 Aug 11;16(8):e0255774. doi: 10.1371/journal.pone.0255774 (PMC8357130; doi:10.1371/journal.pone.0255774)
Supplement: S3 Table — (DOCX) [file pone.0255774.s003.docx]

| **S3 Table.** Overall response rate and response rates for the individual questionnaires at both follow-up time-points. | | | | | | | | | |
| --- | --- | --- | --- | --- | --- | --- | --- | --- | --- |
|  |  |  |  |  | **Overall cohort** | |  | **COVID-19 cohort** | |
|  |  |  | **Overall** |  | **COVID-19** | **non-COVID-19** |  | **ICU** | **non-ICU** |
| **1 month** | |  | 252 |  | 123 | 129 |  | 30 | 93 |
|  | ***IES-R*** |  | 237 (94%) |  | 116 (94%) | 121 (94%) |  | 30 (100%) | 86 (92%) |
|  | ***HADS*** |  | 248 (98%) |  | 121 (98%) | 127 (98%) |  | 30 (100%) | 91 (98%) |
|  | ***EQ-5D*** |  | 249 (99%) |  | 121 (98%) | 128 (99%) |  | 29 (97%) | 92 (99%) |
|  | ***RAND-36*** |  | 242 (96%) |  | 120 (98%) | 122 (95%) |  | 29 (97%) | 91 (98%) |
|  |  |  |  |  |  |  |  |  |  |
| **3 months** | |  | 212 |  | 116 | 96 |  | 33 | 83 |
|  | ***IES-R*** |  | 209 (99%) |  | 116 (100%) | 93 (97%) |  | 33 (100%) | 83 (100%) |
|  | ***HADS*** |  | 208 (98%) |  | 115 (99%) | 93 (97%) |  | 33 (100%) | 82 (99%) |
|  | ***EQ-5D*** |  | 210 (99%) |  | 116 (100%) | 94 (98%) |  | 33 (100%) | 83 (100%) |
|  | ***RAND-36*** |  | 206 (97%) |  | 114 (98%) | 92 (96%) |  | 33 (100%) | 81 (98%) |
