# Supplementary material for: Psychological distress and health-related quality of life in patients after hospitalization during the COVID-19 pandemic: A single-center, observational study
Source: PLoS One. 2021 Aug 11;16(8):e0255774. doi: 10.1371/journal.pone.0255774 (PMC8357130; doi:10.1371/journal.pone.0255774)
Supplement: S4 Table — (DOCX) [file pone.0255774.s004.docx]

| S4 Table. Non-responders’ analysis based on baseline demographics and treatment-related characteristics. | | | | | | |
| --- | --- | --- | --- | --- | --- | --- |
|  | |  | **Responders** |  | **Non-responders** |  |
|  | |  | **(n=294)** |  | **(n=328)** | **p-value** |
| Age, years | |  | 64 (34-88) |  | 66 (25-91) | 0.169 |
| Sex at birth, Female | |  | 102 (36%) |  | 163 (48) | **0.004** |
| Ethnicity | |  |  |  |  |  |
|  | Caucasian |  | 198 (70%) |  | 215 (63%) | >0.05 |
|  | Negroid |  | 10 (4%) |  | 22 (6%) | >0.05 |
|  | Surinamese /Hindustan |  | 21 (7%) |  | 20 (6%) | >0.05 |
|  | Arab, not specified |  | 13 (5%) |  | 21 (6%) | >0.05 |
|  | Turkish |  | 7 (3%) |  | 16 (5%) | >0.05 |
|  | Moroccan |  | 10 (4%) |  | 9 (3%) | >0.05 |
|  | Others |  | 8 (3%) |  | 8 (2%) | >0.05 |
|  | Unknown |  | 15 (5%) |  | 30 (9%) | >0.05 |
| BMI | |  | 27.5 (19.2-43.0) |  | 26.2 (17.6-41.1) | **0.029** |
|  | BMI <25 |  | 51 (18%) |  | 84 (25%) | **0.021** |
|  | BMI 25-30 |  | 64 (23%) |  | 62 (18%) | >0.05 |
|  | BMI >30 |  | 60 (21%) |  | 55 (16%) | >0.05 |
| Comorbidities | |  |  |  |  |  |
|  | Hypertension |  | 83 (38%) |  | 132 (49%) | **0.017** |
|  | Chronic cardiac disease |  | 65 (77%) |  | 78 (23%) | 0.974 |
|  | Chronic pulmonary disease |  | 52 (18%) |  | 74 (22%) | 0.304 |
|  | Asthma |  | 34 (12%) |  | 38 (11%) | 0.761 |
|  | Tuberculosis |  | 2 (2%) |  | 0 (0%) | 0.112 |
|  | Chronic kidney disease |  | 14 (5%) |  | 33 (10%) | 0.026 |
|  | Mild liver disease |  | 6 (2%) |  | 5 (2%) | 0.536 |
|  | Moderate liver disease |  | 1 (1%) |  | 2 (1%) | 0.675 |
|  | Chronic neurological disease |  | 25 (9%) |  | 43 (13%) | 0.129 |
|  | Dementia |  | 7 (3%) |  | 15 (5%) | 0.193 |
|  | Chronic hematologic disease |  | 7 (3%) |  | 12 (4%) | 0.45 |
|  | Diabetes type 1 or 2 |  | 59 (21%) |  | 83 (24%) | 0.336 |
|  | Rheumatologic disorder |  | 20 (7%) |  | 23 (7%) | 0.871 |
|  | Malignant neoplasm |  | 17 (6%) |  | 38 (11%) | **0.024** |
| Total number of comorbidities | |  | 1 (0-4) |  | 2 (0-5) | **0.006** |
| 2 or more comorbidities | |  | 106 (38%) |  | 172 (50%) | **<0.001** |
| Healthcare worker | |  | 17 (6%) |  | 11 (3%) | 0.181 |
| Smoking status | |  |  |  |  |  |
|  | Smoker |  | 25 (9%) |  | 59 (17%) | **0.002** |
|  | Never smoker |  | 145 (51%) |  | 158 (46%) | >0,05 |
|  | Former smoker |  | 94 (33%) |  | 112 (32%) | >0.05 |
|  | Unknown |  | 18 (6%) |  | 12 (4%) | >0.05 |
| Diagnosis COVID-19 | |  | 144 (51%) |  | 111 (33%) | **<0.001** |
| Hospital LOS, days | |  | 5 (1-55) |  | 5 (1-49) | 0.856 |
| ICU admission | |  | 41 (15%) |  | 33 (10%) | 0.060 |
| ICU LOS, days | |  | 16 (0-52) |  | 20 (0-84) | 0.305 |
| SOFA score* | |  | 2 (0-6) |  | 2 (0-6) | 0.201 |
| P/F ratio* | |  | 323 (76-548) |  | 314 (98-560) | 0.599 |
| S/F ratio* | |  | 448 (104-471) |  | 442 (97-476) | 0.952 |
| Need for oxygen therapy | |  | 213 (76%) |  | 253 (74%) | 0.657 |
| Duration of oxygen therapy, days | |  | 6 (1-53) |  | 4 (1-48) | 0.126 |
| Non-invasive ventilation | |  | 13 (5%) |  | 11 (3%) | 0.376 |
| Duration non-invasive ventilation, days | |  | 4 (1-23) |  | 3 (1-12) | 0.633 |
| Invasive ventilation | |  | 35 (12%) |  | 30 (9%) | 0.139 |
| Duration invasive ventilation, days | |  | 14 (1-52) |  | 19 (2-59) | 0.307 |
| Prone positioning | |  | 15 (5%) |  | 18 (5%) | 0.975 |
| Duration prone positioning, days | |  | 5 (1-14) |  | 5 (1-10) | 0.956 |
| Tracheostomy | |  | 14 (5%) |  | 13 (4%) | 0.477 |
| Data are shown as n (%) and median (95% range). Abbreviations: ICU, Intensive care unit; LOS, length of stay; SOFA, Sequential Organ Failure Assessment; P/F ratio, ratio between arterial partial pressure (PaO_2_) to fractional inspired oxygen (FiO_2_); S/F ratio, ratio between peripheral oxygen saturation (SaO_2_) and FiO_2_. * scored the day of first SARS-CoV-2 suspicion. P-value calculated using a Mann Whitney-U Test for continuous variables and using a Fisher exact test for categorical variables. | | | | | | |
