# Supplementary material for: Psychological distress and health-related quality of life in patients after hospitalization during the COVID-19 pandemic: A single-center, observational study
Source: PLoS One. 2021 Aug 11;16(8):e0255774. doi: 10.1371/journal.pone.0255774 (PMC8357130; doi:10.1371/journal.pone.0255774)
Supplement: S5 Table — (DOCX) [file pone.0255774.s005.docx]

| **S5 Table. Comparison of baseline demographics and treatment-related characteristics between full responders and participants who only responded at 1 of 3 months after discharge.** | | | | | | | | | | |
| --- | --- | --- | --- | --- | --- | --- | --- | --- | --- | --- |
|  |  |  | **Full Responders** |  | **Partial Responders (only 1 month)** | *p*-value |  | **Partial Responders (only 3 months)** | *p*-value |  |
| Sample size | |  | 170 |  | 82 |  |  | 42 |  |  |
| Age, years | |  | 64 (37-87) |  | 65 (27-89) | 0.69 |  | 62 (35-92) | 0.38 |  |
| Ethnicity | |  |  |  |  |  |  |  |  |  |
|  | Caucasian |  | 133 (78%) |  | 46 (56%) | **<0.001** |  | 28 (67%) | 0.16 |  |
|  | Black |  | 6 (4%) |  | 9 (4%) | 1.00 |  | 1 (2%) | 1.00 |  |
|  | Surinamese /Hindustan |  | 11 (6%) |  | 9 (11%) | 0.22 |  | 2 (5%) | 1.00 |  |
|  | Arab, not specified |  | 7 (4%) |  | 5 (6%) | 0.53 |  | 1 (2%) | 1.00 |  |
|  | Turkish |  | 1 (1%) |  | 4 (5%) | **0.04** |  | 3 (7%) | **0.03** |  |
|  | Moroccan |  | 2 (1%) |  | 7 (9%) | **0.006** |  | 1 (2%) | 0.49 |  |
|  | Others |  | 2 (1%) |  | 4 (5%) | 0.09 |  | 2 (5%) | 0.18 |  |
|  | Unknown |  | 8 (5%) |  | 4 (5%) | 1.00 |  | 4 (10%) | 0.26 |  |
| Sex at birth, Female | |  | 57 (34%) |  | 34 (41%) | 0.26 |  | 15 (36%) | 0.86 |  |
| Body Mass Index (BMI) * | |  | 26.5 (19.1-40.7) |  | 27.3 (20.1-43.2) | 0.62 |  | 31 (20-46) | 0.02 |  |
|  | BMI <25 |  | 31 (31%) |  | 17 (30%) | 0.73 |  | 5 (21%) | 0.49 |  |
|  | BMI 25-30 |  | 39 (39%) |  | 20 (36%) | 0.87 |  | 6 (25%) | 0.29 |  |
|  | BMI >30 |  | 30 (30%) |  | 19 (34%) | 0.31 |  | 13 (54%) | 0.08 |  |
| Comorbidities | |  |  |  |  |  |  |  |  |  |
|  | Hypertension |  | 50 (38%) |  | 21 (34%) | 0.63 |  | 13 (43%) | 0.68 |  |
|  | Chronic cardiac disease |  | 38 (22%) |  | 17 (21%) | 0.87 |  | 12 (29%) | 0.42 |  |
|  | Chronic pulmonary disease |  | 29 (17%) |  | 16 (19%) | 0.73 |  | 9 (21%) | 0.51 |  |
|  | Asthma |  | 27 (16%) |  | 4 (5%) | **0.02** |  | 8 (19%) | 0.65 |  |
|  | Tuberculosis |  | 1 (1%) |  | 1 (2%) | 1.00 |  | 0 (0%) | 1.00 |  |
|  | Chronic kidney disease |  | 8 (5%) |  | 5 (6%) | 0.76 |  | 3 (7%) | 0.46 |  |
|  | Mild liver disease |  | 2 (1%) |  | 4 (5%) | 0.09 |  | 0 (0%) | 1.00 |  |
|  | Moderate liver disease |  | 1 (1%) |  | 0 (0%) | 1.00 |  | 0 (0%) | 1.00 |  |
|  | Chronic neurological disease |  | 10 (6%) |  | 10 (13%) | 0.052 |  | 4 (10%) | 0.48 |  |
|  | Dementia |  | 3 (2%) |  | 3 (4%) | 0.39 |  | 1 (2%) | 1.00 |  |
|  | Chronic hematologic disease |  | 3 (2%) |  | 2 (2%) | 0.66 |  | 2 (5%) | 0.26 |  |
|  | Diabetes type I or II |  | 30 (18%) |  | 22 (27%) | 0.13 |  | 9 (22%) | 0.51 |  |
|  | Rheumatologic disorder |  | 11 (6%) |  | 6 (7%) | 0.79 |  | 4 (10%) | 0.50 |  |
|  | Malignant neoplasm |  | 11 (6%) |  | 4 (5%) | 0.78 |  | 2 (5%) | 1.00 |  |
| Total number of comorbidities | |  | 1 (0-4) |  | 1 (0-5) | 0.52 |  | 1 (0-4) | 0.12 |  |
|  | 2 or more comorbidities |  | 57 (34%) |  | 33 (40%) | 0.33 |  | 20 (48%) | 0.11 |  |
| Psychiatric problems in past 5 years ** | |  | 22 (13%) |  | N/A | N/A |  | 4 (12%) | 1.00 |  |
| Psychological treatment ** | |  |  |  | N/A | N/A |  |  |  |  |
|  | Yes, psychologist |  | 7 (4%) |  | N/A | N/A |  | 3 (7%) | 0.42 |  |
|  | Yes, psychiatrist |  | 3 (2%) |  | N/A | N/A |  | 1 (2%) | 1.00 |  |
|  | Yes, psychologist and psychiatrist |  | 6 (4%) |  | N/A | N/A |  | 0 (0%) | 0.60 |  |
|  | Yes, medication |  | 10 (6%) |  | N/A | N/A |  | 1 (3%) | 1.00 |  |
| Smoking | |  |  |  |  |  |  |  |  |  |
|  | Yes |  | 13 (8%) |  | 8 (10%) | 0.63 |  | 7 (17%) | 0.08 |  |
|  | Never smoker |  | 94 (55%) |  | 40 (49%) | 0.35 |  | 15 (36%) | **0.03** |  |
|  | Former smoker |  | 53 (21%) |  | 31 (38%) | 0.32 |  | 15 (36%) | 0.58 |  |
|  | Unknown |  | 10 (6%) |  | 3 (4%) | 0.56 |  | 5 (12%) | 0.18 |  |
| Educational level *** | |  |  |  |  |  |  |  |  |  |
|  | Elementary school |  | 24 (14%) |  | 22 (27%) | **0.02** |  | N/A | N/A |  |
|  | High school |  | 32 (19%) |  | 17 (21%) | 0.73 |  | N/A | N/A |  |
|  | Intermediate vocational education |  | 63 (37%) |  | 26 (32%) | 0.48 |  | N/A | N/A |  |
|  | Bachelor’s degree |  | 26 (15%) |  | 14 (17%) | 0.71 |  | N/A | N/A |  |
|  | Master’s degree |  | 24 (14%) |  | 2 (2%) | **0.003** |  | N/A | N/A |  |
| Working/employed before admission *** | |  | 69 (41%) |  | 23 (28%) | 0.07 |  | N/A | N/A |  |
| Work hours before admission *** | |  | 36 (8-49) |  | 36 (18-61) | 0.81 |  | N/A | N/A |  |
| Healthcare worker | |  | 10 (7%) |  | 6 (9%) | 0.78 |  | 2 (6%) | 1.00 |  |
| Cause of admission | |  |  |  |  |  |  |  |  |  |
|  | COVID-19 |  | 93 (55%) |  | 30 (37%) | **0.007** |  | 23 (55%) | 1.00 |  |
|  | Lower respiratory tract infection |  | 17 (10%) |  | 8 (10%) | 1.00 |  | 3 (7%) | 0.77 |  |
|  | Exacerbation asthma/COPD |  | 11 (6%) |  | 10 (12%) | 0.15 |  | 4 (10%) | 0.50 |  |
|  | Pulmonary malignancy |  | 3 (2%) |  | 1 (1%) | 1.00 |  | 0 (0%) | 1.00 |  |
|  | Other malignancy |  | 3 (2%) |  | 0 (0%) | 0.55 |  | 1 (2%) | 1.00 |  |
|  | Pulmonary embolism |  | 1 (1%) |  | 1 (1%) | 0.55 |  | 1 (2%) | 0.36 |  |
|  | Other respiratory disease |  | 3 (2%) |  | 3 (4%) | 0.39 |  | 3 (7%) | 0.09 |  |
|  | Cardiac pathology |  | 14 (8%) |  | 9 (11%) | 0.49 |  | 1 (2%) | 0.31 |  |
|  | Gastro-intestinal pathology |  | 10 (6%) |  | 6 (7%) | 0.78 |  | 0 (0%) | 0.22 |  |
|  | Urogenital pathology |  | 8 (5%) |  | 6 (7%) | 0.39 |  | 2 (5%) | 1.00 |  |
|  | Other |  | 7 (4%) |  | 8 (10%) | 0.09 |  | 4 (10%) | 0.23 |  |
| Treatment restrictions (at admission) | |  |  |  |  |  |  |  |  |  |
|  | Code 1, No restrictions |  | 120 (74%) |  | 49 (68%) | 0.34 |  | 25 (69%) | 0.54 |  |
|  | Code 2, DNR |  | 8 (5%) |  | 5 (7%) | 0.55 |  | 3 (8%) | 0.43 |  |
|  | Code 3, DNR, DNI |  | 11 (7%) |  | 7 (10%) | 0.44 |  | 4 (11%) | 0.48 |  |
|  | Code 4, DNR, DNI, no ICU admission |  | 22 (14%) |  | 11 (15%) | 0.84 |  | 4 (11%) | 0.79 |  |
|  | Code 5, abstain supportive care |  | 0 (0%) |  | 0 (0%) | N/A |  | 0 (0%) | N/A |  |
|  | Unknown |  | 0 (0%) |  | 0 (0%) | N/A |  | 0 (0%) | N/A |  |
| Treatment restrictions (before discharge) | |  |  |  |  |  |  |  |  |  |
|  | Code 1, No restrictions |  | 129 (76%) |  | 58 (71%) | 0.44 |  | 30 (71%) | 0.55 |  |
|  | Code 2, DNR |  | 8 (5%) |  | 4 (5%) | 1.00 |  | 0 (0%) | 0.36 |  |
|  | Code 3, DNR, DNI |  | 9 (5%) |  | 6 (7%) | 0.57 |  | 4 (10%) | 0.29 |  |
|  | Code 4, DNR, DNI, no ICU admission |  | 24 (14%) |  | 13 (16%) | 0.71 |  | 7 (17%) | 0.63 |  |
|  | Code 5, abstain supportive care |  | 0 (0%) |  | 0 (0%) | N/A |  | 0 (0%) | N/A |  |
|  | Unknown |  | 0 (0%) |  | 1 (1%) | 0.33 |  | 1 (2%) | 0.20 |  |
| Hospital length of stay, days | |  | 4 (1-46) |  | 4 (1-46) | 0.68 |  | 6 (1-67) | 0.16 |  |
| Admitted to the ICU | |  | 25 (15%) |  | 7 (9%) | 0.23 |  | 10 (24%) | 0.17 |  |
| ICU length of stay, days | |  | 16 (0-48) |  | 25 (3-46) | 0.31 |  | 13 (8-47) | 1.00 |  |
| SOFA score **** | |  | 2 (0-6) |  | 2 (0-6) | 0.51 |  | 2 (0-6) | 0.47 |  |
| P/F ratio **** | |  | 328 (102-524) |  | 324 (100-542) | 0.58 |  | 319 (69-567) | 0.36 |  |
| S/F ratio **** | |  | 448 (132-471) |  | 445 (100-476) | 0.76 |  | 452 (93-471) | 0.64 |  |
| Received oxygen therapy | |  | 127 (75%) |  | 62 (76%) | 1.00 |  | 33 (79%) | 0.69 |  |
| Duration of oxygen therapy, days | |  | 6 (10-45) |  | 4 (1-48) | 0.62 |  | 12 (1-47) | 0.25 |  |
| Received non-invasive ventilation (NIV) | |  | 8 (5%) |  | 4 (5%) | 1.00 |  | 1 (2%) | 0.69 |  |
| Duration of NIV, days | |  | 4 (1-10) |  | 8 (1-22) | 0.59 |  | 4 (4-4) | 1.00 |  |
| Received invasive ventilation | |  | 20 (12%) |  | 7 (9%) | 0.52 |  | 9 (21%) | 0.13 |  |
| Duration of invasive ventilation, days | |  | 14 (1-35) |  | 25 (10-45) | 0.17 |  | 12 (8-47) | 0.91 |  |
| Ventilated in prone position | |  | 9 (5%) |  | 4 (5%) | 1.00 |  | 3 (7%) | 0.71 |  |
| Duration of prone positioning, days | |  | 5 (1-11) |  | 4 (2-13) | 0.94 |  | 5 (1-7) | 0.64 |  |
| Received a tracheostomy | |  | 7 (4%) |  | 4 (5%)) | 0.75 |  | 3 (7%) | 0.42 |  |
| Died during follow-up | |  | 0 (0%) |  | 4 (5%) | **0.01** |  | 0 (0%) | 1.00 |  |
| Data are shown as n (%) and median (95% range). Abbreviations: ICU, Intensive care unit; SOFA, Sequential Organ Failure Assessment; P/F ratio, ratio between arterial partial pressure (PaO2) to fractional inspired oxygen (FiO2); S/F ratio, ratio between peripheral oxygen saturation (SaO2) and FiO2. *P*-values were calculated using a Mann Whitney-U Test for continuous variables and using a Fisher’s Exact test for categorical variables. * BMI of 116 patients was not available. ** Results regarding psychological history are derived from the questionnaire 3 months after discharge. *** Results regarding educational level and employment status after hospitalization are derived from the questionnaires 1 month after discharge. **** Scored the day of first SARS-CoV-2 suspicion. Non-invasive ventilation was defined as use of CPAP or BIPAP; Use of high flow nasal cannula was not included | | | | | | | | | | |
