# Supplementary material for: Psychological distress and health-related quality of life in patients after hospitalization during the COVID-19 pandemic: A single-center, observational study
Source: PLoS One. 2021 Aug 11;16(8):e0255774. doi: 10.1371/journal.pone.0255774 (PMC8357130; doi:10.1371/journal.pone.0255774)
Supplement: S6 Table — (DOCX) [file pone.0255774.s006.docx]

| **S6 Table.** Comparison psychological and HRQoL outcomes between full responders and participants who only responded at 1 of 3 months after discharge. | | | | | | | | | |  |
| --- | --- | --- | --- | --- | --- | --- | --- | --- | --- | --- |
|  |  |  | **Full Responders** |  | **Partial Responders (only 1 month)** | *p*-value |  | **Partial Responders (only 3 months)** | *p*-value | |
|  | 1 month |  | n = 170 |  | 82 |  |  | N/A |  | |
|  | 3 months |  | n = 170 |  | N/A |  |  | 42 |  | |
| **PTSD** |  |  |  |  |  |  |  |  |  | |
| ***Severity, median (95% range)*** | |  |  |  |  |  |  |  |  | |
|  | 1 month |  | 8 (0-45) |  | 10 (0-55) | 0.23^1^ |  | N/A | N/A | |
|  | 3 months |  | 6 (0-44) |  | N/A | N/A |  | 8 (0-36) | 0.30^2^ | |
| ***Prevalence, n (%)*** | |  |  |  |  |  |  |  |  | |
|  | 1 month |  | 23 (10%) |  | 16 (7%) | 0.31^2^ |  | N/A | N/A | |
|  | 3 months |  | 18 (9%) |  | N/A | N/A |  | 10 (5%) | 0.06^2^ | |
|  |  |  |  |  |  |  |  |  |  | |
| **Anxiety** |  |  |  |  |  |  |  |  |  | |
| ***Severity, median (95% range)*** | |  |  |  |  |  |  |  |  | |
|  | 1 month |  | 4 (0-14) |  | 6 (0-20) | 0.03^3^ |  | N/A | N/A | |
|  | 3 months |  | 3 (0-15) |  | N/A | N/A |  | 5 (0-13) | 0.31^2^ | |
| ***Prevalence, n (%)*** | |  |  |  |  |  |  |  |  | |
|  | 1 month |  | 41 (17%) |  | 31 (13%) | 0.08^1^ |  | N/A | N/A | |
|  | 3 months |  | 32 (15%) |  | N/A | N/A |  | 10 (5%) | 0.61^2^ | |
|  |  |  |  |  |  |  |  |  |  | |
| **Depression** |  |  |  |  |  |  |  |  |  | |
| ***Severity, median (95% range)*** | |  |  |  |  |  |  |  |  | |
|  | 1 month |  | 4 (0-14) |  | 6 (0-18) | 0.13^3^ |  | N/A | N/A | |
|  | 3 months |  | 3 (0-16) |  | N/A | N/A |  | 5 (0-16) | 0.22^2^ | |
| ***Prevalence, n (%)*** | |  |  |  |  |  |  |  |  | |
|  | 1 month |  | 48 (19%) |  | 31 (13%) | 0.42 |  | N/A | N/A | |
|  | 3 months |  | 38 (18%) |  | N/A | N/A |  | 12 (6%) | 0.33^5^ | |
|  |  |  |  |  |  |  |  |  |  | |
| **Overall HRQoL, median (95% range)** | | | |  |  |  |  |  |  | |
|  | 1 month |  | 0.75  (0.00-1.00) |  | 0.70  (-0.29 - 1.00) | 0.02^3^ |  | N/A | N/A | |
|  | 3 months |  | 0.79  (0.09-1.00) |  | N/A | N/A |  | 0.73  (0.03-1.00) | 0.12^2^ | |
|  |  |  |  |  |  |  |  |  |  | |
| **Perceived health state, median (95% range)** | | | |  |  |  |  |  |  | |
|  | 1 month |  | 65 (10-95) |  | 63 (19-96) | 0.77^4^ |  | N/A | N/A | |
|  | 3 months |  | 75 (9-95) |  | N/A | N/A |  | 65 (10-100) | 0.15^2^ | |
|  |  |  |  |  |  |  |  |  |  | |
| **Mental HRQoL, median (95% range)** | | | |  |  |  |  |  |  | |
|  | 1 month |  | 40 (17-63) |  | 39 (14-61) | 0.62^1^ |  | N/A | N/A | |
|  | 3 months |  | 49 (19-64) |  | N/A | N/A |  | 43 (20-63) | 0.41^2^ | |
|  |  |  |  |  |  |  |  |  |  | |
| **Physical HRQoL, median (95% range)** | | | |  |  |  |  |  |  | |
|  | 1 month |  | 37 (18-56) |  | 36 (19-55) | 0.58^4^ |  | N/A | N/A | |
|  | 3 months |  | 39 (18-58) |  | N/A | N/A |  | 38 (13-59) | 0.31^2^ | |
| Descriptive statistics of the psychological distress and HRQoL outcomes, stratified by response status (full responders vs. partial responder who only responded at either 1 or 3 months). Severity of PTSD, anxiety, and depression were expressed as the IES-R, HADS anxiety, and HADS depression sum scores, respectively. Prevalence of probable PTSD, anxiety, and depression was defined as the proportion of patients scoring above the cut-off. Overall HRQoL was expressed as the EQ-5D TTO score, the perceived health state as the EQ-5D VAS score, mental HRQoL as the MCS-36, and physical HRQoL as the PCS-36. Differences between full and partial responders were analyzed using simple linear (for continuous outcomes) and logistic (for categorical outcomes) regression models. ^1^ Adjusted for ethnicity and educational level, ^2^ adjusted for ethnicity, ^3^ adjusted for ethnicity, educational level, and COVID-19 diagnosis, ^4^ not adjusted.. Abbreviations: CI, confidence interval; COVID-19, coronavirus disease 2019; ICU, intensive care unit; OR, odds ratio; PTSD, post-traumatic stress disorder | | | | | | | | | | |
