# Supplementary material for: Psychological distress and health-related quality of life in patients after hospitalization during the COVID-19 pandemic: A single-center, observational study
Source: PLoS One. 2021 Aug 11;16(8):e0255774. doi: 10.1371/journal.pone.0255774 (PMC8357130; doi:10.1371/journal.pone.0255774)
Supplement: S7 Table — (DOCX) [file pone.0255774.s007.docx]

| **S7 Table.** Non-responders’ analysis based on psychological outcomes and health-related quality of life. | | | | | |
| --- | --- | --- | --- | --- | --- |
|  |  | **Responders** |  | **Non-responders** | ***p*-value** |
|  |  | n = 212 |  | 12 |  |
| **PTSD** |  |  |  |  |  |
| ***Severity, median (95% range)*** |  | 7 (0-44) |  | 6 (0-42) | 0.22^1^ |
| ***Prevalence, n (%)*** |  | 28 (13%) |  | 3 (27%) | 0.18^1^ |
|  |  |  |  |  |  |
| **Anxiety** |  |  |  |  |  |
| ***Severity, median (95% range)*** |  | 4 (0-15) |  | 6 (0-13) | 0.27^1^ |
| ***Prevalence, n (%)*** |  | 42 (20%) |  | 3 (27%) | 0.57^2^ |
|  |  |  |  |  |  |
| **Depression** |  |  |  |  |  |
| ***Severity, median (95% range)*** |  | 4 (0-16) |  | 4 (0-12) | 0.92^1^ |
| ***Prevalence, n (%)*** |  | 50 (24%) |  | 2 (18%) | 0.66^2^ |
|  |  |  |  |  |  |
| **Overall HRQoL, median (95% range)** |  | 0.78 (0.07-1.00) |  | 0.70 (0.20-1.00) | 0.65^3^ |
|  |  |  |  |  |  |
| **Perceived health state, median (95% range)** |  | 73 (9-95) |  | 65 (14-94) | 0.85^3^ |
|  |  |  |  |  |  |
| **Mental HRQoL, median (95% range)** |  | 48 (19-64) |  | 34 (21-60) | 0.15^1^ |
|  |  |  |  |  |  |
| **Physical HRQoL, median (95% range)** |  | 39 (17-59) |  | 42 (17-59) | 0.33^3^ |
| Descriptive statistics of the psychological distress and HRQoL outcomes, stratified by response status (responders vs. non-responders). Severity of PTSD, anxiety, and depression were expressed as the IES-R, HADS anxiety, and HADS depression sum scores, respectively. Prevalence of probable PTSD, anxiety, and depression was defined as the proportion of patients scoring above the cut-off. Overall HRQoL was expressed as the EQ-5D TTO score, the perceived health state as the EQ-5D VAS score, mental HRQoL as the MCS-36, and physical HRQoL as the PCS-36. Differences between full and partial responders were analyzed using simple linear (for continuous outcomes) and logistic (for categorical outcomes) regression models. ^1^ Adjusted for gender, ^2^ not adjusted, ^3^ adjusted for gender and COVID-19 diagnosis. Abbreviations: CI, confidence interval; COVID-19, coronavirus disease 2019; ICU, intensive care unit; OR, odds ratio; PTSD, post-traumatic stress disorder | | | | | |
