# Supplementary material for: Psychological distress and health-related quality of life in patients after hospitalization during the COVID-19 pandemic: A single-center, observational study
Source: PLoS One. 2021 Aug 11;16(8):e0255774. doi: 10.1371/journal.pone.0255774 (PMC8357130; doi:10.1371/journal.pone.0255774)
Supplement: S8 Table — (DOCX) [file pone.0255774.s008.docx]

| **S8 Table.** Baseline demographics and treatment-related characteristics of study participants. | | | | | | | | | | | |  |
| --- | --- | --- | --- | --- | --- | --- | --- | --- | --- | --- | --- | --- |
|  |  |  |  |  | **Overall cohort** | | |  | **COVID-19 cohort** | | | |
|  |  |  | **Overall** |  | **COVID-19** | **non-COVID-19** | *p*-value |  | **ICU** | **non-ICU** | *p*-value | |
| Sample size | |  | 294 |  | 146 | 148 |  |  | 40 | 106 |  | |
| Age, years | |  | 64 (33-88) |  | 61 (35-85) | 69 (31-89) | <0.001 |  | 62 (36-74) | 60 (33-86) | 0.37 | |
| Ethnicity | |  |  |  |  |  |  |  |  |  |  | |
|  | Caucasian |  | 207 (70%) |  | 92 (63%) | 115 (78%) | <0.01 |  | 22 (55%) | 70 (66%) | 0.25 | |
|  | Black |  | 10 (3%) |  | 8 (5%) | 2 (1%) | 0.06 |  | 3 (8%) | 5 (5%) | 0.68 | |
|  | Surinamese /Hindustan |  | 22 (7%) |  | 15 (10%) | 7 (5%) | 0.08 |  | 7 (18%) | 8 (8%) | 0.12 | |
|  | Arab, not specified |  | 13 (4%) |  | 6 (4%) | 7 (5%) | 1.00 |  | 1 (3%) | 5 (5%) | 1.00 | |
|  | Turkish |  | 8 (3%) |  | 5 (3%) | 3 (2%) | 0.50 |  | 0 (0%) | 5 (5%) | 0.32 | |
|  | Moroccan |  | 10 (3%) |  | 8 (5%) | 2 (1%) | 0.06 |  | 4 (10%) | 4 (4%) | 0.22 | |
|  | Others |  | 8 (3%) |  | 5 (3%) | 3 (2%) | 0.50 |  | 1 (3%) | 4 (4%) | 1.00 | |
|  | Unknown |  | 16 (5%) |  | 7 (5) | 9 (6%) | 0.80 |  | 2 (5%) | 5 (5%) | 1.00 | |
| Sex at birth, Female | |  | 106 (36%) |  | 52 (36%) | 54 (36%) | 0.90 |  | 13 (33%) | 39 (37%) | 0.70 | |
| Body Mass Index (BMI) * | |  | 27.4 (19.3-43.2) |  | 28.0 (20.4-41.8) | 26.5 (18.9-43.1) | 0.30 |  | 28.5 (22.7-41.9) | 27.9 (19.7-39.5) | 0.35 | |
|  | BMI <25 |  | 53 (29%) |  | 19 (26%) | 34 (32%) | 0.03 |  | 6 (19%) | 13 (31%) | 0.78 | |
|  | BMI 25-30 |  | 62 (34%) |  | 28 (38%) | 34 (32%) | 0.16 |  | 14 (44%) | 14 (33%) | 0.04 | |
|  | BMI >30 |  | 65 (36%) |  | 27 (36%) | 38 (36%) | 0.48 |  | 12 (38%) | 15 (36%) | <0.01 | |
| Comorbidities | |  |  |  |  |  |  |  |  |  |  | |
|  | Hypertension |  | 84 (38%) |  | 43 (36%) | 41 (39%) | 0.78 |  | 9 (28%) | 34 (40%) | 0.29 | |
|  | Chronic cardiac disease |  | 67 (23%) |  | 18 (12%) | 49 (33%) | <0.001 |  | 3 (8%) | 15 (14%) | 0.40 | |
|  | Chronic pulmonary disease |  | 54 (18%) |  | 10 (7%) | 44 (29%) | <0.001 |  | 3 (8%) | 7 (7%) | 1.00 | |
|  | Asthma |  | 39 (13%) |  | 18 (12%) | 21 (14%) | 0.73 |  | 5 (13%) | 13 (12%) | 1.00 | |
|  | Tuberculosis |  | 2 (2%) |  | 0 (0%) | 2 (2%) | 1.00 |  | 0 (0%) | 0 (0%) | 1.00 | |
|  | Chronic kidney disease |  | 16 (5%) |  | 3 (2%) | 13 (9%) | 0.02 |  | 1 (3%) | 2 (2%) | 1.00 | |
|  | Mild liver disease |  | 6 (2%) |  | 2 (1%) | 4 (3%) | 0.68 |  | 0 (0%) | 2 (2%) | 1.00 | |
|  | Moderate liver disease |  | 1 (0%) |  | 0 (0%) | 1 (0%) | 1.00 |  | 0 (0%) | 0 (0%) | 1.00 | |
|  | Chronic neurological disease |  | 25 (9%) |  | 9 (6%) | 16 (11%) | 0.21 |  | 2 (5%) | 7 (7%) | 1.00 | |
|  | Dementia |  | 7 (2%) |  | 3 (2%) | 4 (3%) | 1.00 |  | 0 (0%) | 3 (3%) | 0.56 | |
|  | Chronic hematologic disease |  | 7 (2%) |  | 3 (2%) | 4 (3%) | 1.00 |  | 0 (0%) | 3 (3%) | 0.56 | |
|  | Diabetes type I or II |  | 61 (21%) |  | 26 (18%) | 35 (24%) | 0.25 |  | 7 (18%) | 19 (18%) | 1.00 | |
|  | Rheumatologic disorder |  | 21 (7%) |  | 5 (3%) | 16 (11%) | 0.02 |  | 1 (3%) | 4 (4%) | 1.00 | |
|  | Malignant neoplasm |  | 17 (6%) |  | 4 (3%) | 13 (9%) | 0.04 |  | 1 (3%) | 3 (3%) | 1.00 | |
| Total number of comorbidities | |  | 1 (0-4) |  | 1 (0-3) | 2 (0-4) | <0.001 |  | 1 (0-3) | 1 (0-3) | 0.16 | |
|  | 2 or more comorbidities |  | 110 (37%) |  | 35 (24%) | 75 (51%) | <0.001 |  | 8 (20%) | 27 (25%) | 0.66 | |
| Psychiatric problems in past 5 years ** | |  | 27 (13%) |  | 12 (11%) | 15 (16%) | 0.41 |  | 3 (12%) | 9 (11%) | 1.00 | |
| Psychological treatment ** | |  |  |  |  |  |  |  |  |  |  | |
|  | Yes, psychologist |  | 10 (3%) |  | 5 (3%) | 5 (3%) | 1.00 |  | 3 (8%) | 2 (2%) | 0.13 | |
|  | Yes, psychiatrist |  | 4 (1%) |  | 3 (2%) | 1 (1%) | 0.37 |  | 1 (3%) | 2 (2%) | 1.00 | |
|  | Yes, psychologist and psychiatrist |  | 6 (2%) |  | 3 (2%) | 3 (2%) | 1.00 |  | 0 (0%) | 3 (3%) | 0.56 | |
|  | Yes, medication |  | 12 (6%) |  | 6 (6%) | 6 (7%) | 0.77 |  | 1 (4%) | 5 (6%) | 1.00 | |
| Smoking | |  |  |  |  |  |  |  |  |  |  | |
|  | Yes |  | 28 (10%) |  | 5 (3%) | 23 (16%) | <0.001 |  | 2 (5%) | 3 (3%) | 0.61 | |
|  | Never smoker |  | 149 (51%) |  | 95 (65%) | 54 (36%) | <0.001 |  | 28 (70%) | 67 (63%) | 0.56 | |
|  | Former smoker |  | 99 (34%) |  | 39 (27%) | 60 (41%) | 0.01 |  | 7 (18%) | 32 (30%) | 0.15 | |
|  | Unknown |  | 18 (6%) |  | 7 (5%) | 11 (7%) | 0.47 |  | 3 (8%) | 4 (4%) | 0.39 | |
| Educational level *** | |  |  |  |  |  |  |  |  |  |  | |
|  | Elementary school |  | 46 (18%) |  | 21 (17%) | 25 (19%) | 0.75 |  | 4 (14%) | 17 (18%) | 0.78 | |
|  | High school |  | 49 (20%) |  | 17 (14%) | 43 (25%) | 0.04 |  | 6 (21%) | 11 (12%) | 0.24 | |
|  | Intermediate vocational education |  | 89 (40%) |  | 44 (36%) | 45 (35%) | 0.89 |  | 10 (34%) | 54 (37%) | 1.00 | |
|  | Bachelor’s degree |  | 40 (16%) |  | 20 (17%) | 20 (16%) | 0.86 |  | 4 (14%) | 16 (17%) | 0.78 | |
|  | Master’s degree |  | 26 (10%) |  | 19 (16%) | 7 (5%) | 0.01 |  | 5 (17%) | 14 (15%) | 0.78 | |
| Working/employed before admission^‡^ | |  | 92 (37%) |  | 59 (49%) | 33 (26%) | <0.001 |  | 12 (41%) | 47 (51%) | 0.40 | |
| Work hours before admission | |  | 36 (9-59) |  | 36 (11-54) | 35 (13-57) | 0.21 |  | 37 (11-40) | 36 (15-59) | 0.75 | |
| Healthcare worker | |  | 18 (8%) |  | 13 (11%) | 5 (4%) | 0.08 |  | 3 (10%) | 10 (11%) | 1.00 | |
| Cause of admission | |  |  |  |  |  |  |  |  |  |  | |
|  | COVID-19 |  | 146 (50%) |  | 146 (100%) | 0 (0%) | N/A |  | 40 (100%) | 106 (100%) | N/A | |
|  | Lower respiratory tract infection |  | 28 (10%) |  | (0%) | 28 (19%) | N/A |  | (0%) | (0%) | N/A | |
|  | Exacerbation asthma/COPD |  | 25 (9%) |  | (0%) | 25 (17%) | N/A |  | (0%) | (0%) | N/A | |
|  | Pulmonary malignancy |  | 4 (1%) |  | (0%) | 4 (3%) | N/A |  | (0%) | (0%) | N/A | |
|  | Other malignancy |  | 4 (1%) |  | (0%) | 4 (3%) | N/A |  | (0%) | (0%) | N/A | |
|  | Pulmonary embolism |  | 3 (1%) |  | (0%) | 3 (2%) | N/A |  | (0%) | (0%) | N/A | |
|  | Other respiratory disease |  | 9 (3%) |  | (0%) | 9 (6%) | N/A |  | (0%) | (0%) | N/A | |
|  | Cardiac pathology |  | 24 (8%) |  | (0%) | 24 (16%) | N/A |  | (0%) | (0%) | N/A | |
|  | Gastro-intestinal pathology |  | 16 (5%) |  | (0%) | 16 (11%) | N/A |  | (0%) | (0%) | N/A | |
|  | Urogenital pathology |  | 16 (5%) |  | (0%) | 16 (11%) | N/A |  | (0%) | (0%) | N/A | |
|  | Other |  | 19 (6%) |  | (0%) | 19 (13%) | N/A |  | (0%) | (0%) | N/A | |
| Treatment restrictions (at admission) | |  |  |  |  |  |  |  |  |  |  | |
|  | Code 1, No restrictions |  | 194 (72%) |  | 113 (88%) | 81 (57%) | <0.001 |  | 40 (100%) | 80 (16%) | 0.01 | |
|  | Code 2, DNR |  | 16 (6%) |  | 4 (3%) | 12 (9%) | 0.07 |  | 0 (0%) | 4 (4%) | 0.57 | |
|  | Code 3, DNR, DNI |  | 22 (8%) |  | 5 (4%) | 17 (12%) | 0.02 |  | 0 (0%) | 5 (5%) | 0.33 | |
|  | Code 4, DNR, DNI, no ICU admission |  | 37 (14%) |  | 6 (5%) | 31 (22%) | <0.001 |  | 0 (0%) | 6 (6%) | 0.34 | |
|  | Code 5, abstain supportive care |  | 0 (0%) |  | 0 (0%) | 0 (0%) | 1.00 |  | 0 (0%) | 0 (0%) | 1.00 | |
|  | Unknown |  | 0 (0%) |  | 0 (0%) | 0 (0%) | 1.00 |  | 0 (0%) | 0 (0%) | 1.00 | |
| Treatment restrictions (before discharge) | |  |  |  |  |  |  |  |  |  |  | |
|  | Code 1, No restrictions |  | 217 (74%) |  | 128 (88%) | 89 (60%) | <0.001 |  | 38 (95%) | 90 (85%) | 0.16 | |
|  | Code 2, DNR |  | 12 (4%) |  | 3 (2%) | 9 (6%) | 0.14 |  | 1 (3%) | 2 (2%) | 1.00 | |
|  | Code 3, DNR, DNI |  | 19 (6%) |  | 5 (3%) | 14 (9%) | 0.06 |  | 0 (0%) | 5 (5%) | 0.32 | |
|  | Code 4, DNR, DNI, no ICU admission |  | 44 (15%) |  | 9 (6%) | 35 (24%) | <0.001 |  | 0 (0%) | 9 (8%) | 0.11 | |
|  | Code 5, abstain supportive care |  | 0 (0%) |  | 0 (0%) | 0 (0%) | 1.00 |  | 0 (0%) | 0 (0%) | 1.00 | |
|  | Unknown |  | 2 (1%) |  | 1 (1%) | 1 (1%) | 1.00 |  | 1 (3%) | 0 (0%) | 0.27 | |
| Hospital length of stay, days | |  | 4 (1-52) |  | 5 (1-60) | 4 (1-23) | <0.001 |  | 28 (9-69) | 4 (1-18) | <0.001 | |
| Admitted to the ICU | |  | 42 (14%) |  | 40 (27%) | 2 (1%) | <0.001 |  | 40 (100%) | 0 (0%) | N/A | |
| ICU length of stay, days | |  | 16 (0-52) |  | 16 (0-52) | 1 (1-2) | 0.01 |  | 16 (0-52) | N/A | N/A | |
| SOFA score **** | |  | 2 (0-6) |  | 2 (0-6) | 2 (0-6) | 0.04 |  | 3 (1-8) | 2 (0-5) | <0.001 | |
| P/F ratio **** | |  | 324 (85-550) |  | 314 (74-487) | 333 (122-564) | 0.01 |  | 267 (71-366) | 335 (177-519) | <0.001 | |
| S/F ratio **** | |  | 448 (106-471) |  | 443 (102-467) | 450 (143-473) | 0.08 |  | 392 (94-462) | 448 (277-467) | <0.001 | |
| Received oxygen therapy | |  | 222 (76%) |  | 135 (92%) | 87 (59%) | <0.001 |  | 40 (100%) | 95 (90%) | 0.04 | |
| Duration of oxygen therapy, days | |  | 6 (1-49) |  | 12 (1-54) | 3 (1-12) | <0.001 |  | 19 (8-56) | 4 (1-15) | <0.001 | |
| Received non-invasive ventilation (NIV) | |  | 13 (4%) |  | 9 (6%) | 4 (3%) | 0.17 |  | 5 (13%) | 4 (4%) | 0.06 | |
| Duration of NIV, days | |  | 4 (1-21) |  | 6 (1-21) | 1 (1-10) | 0.20 |  | 2 (1-22) | 6 (4-6) | 0.62 | |
| Received invasive ventilation | |  | 42 (14%) |  | 40 (27%) | 2 (1%) | <0.001 |  | 40 (100%) | 0 (0%) | N/A | |
| Duration of invasive ventilation, days | |  | 14 (2-48) |  | 14 (4-48) | 2 (1-2) | 0.02 |  | 14 (4-48) | N/A | N/A | |
| Ventilated in prone position | |  | 16 (5%) |  | 16 (11%) | 0 (0%) | <0.001 |  | 16 (40%) | N/A | N/A | |
| Duration of prone positioning, days | |  | 5 (1-13) |  | 5 (1-13) | N/A | N/A |  | 5 (1-3) | N/A | N/A | |
| Received a tracheostomy | |  | 14 (5%) |  | 14 (10%) | 0 (0%) | <0.001 |  | 14 (35%) | 0 (0%) | <0.001 | |
| Died during follow-up | |  | 4 (1%) |  | 0 (0%) | 4 (3%) | 0.12 |  | 0 (0%) | 0 (0%) | 1.00 | |
| Data are shown as n (%) and median (95% range). Patients were stratified based on SARS-CoV2 PCR; COVID-19 and a non-COVID-19. COVID-19 patients were stratified based on necessity for intensive care treatment; COVID-19 ICU and COVID-19 non-ICU. Abbreviations: ICU, Intensive care unit; SOFA, Sequential Organ Failure Assessment; P/F ratio, ratio between arterial partial pressure (PaO2) to fractional inspired oxygen (FiO2); S/F ratio, ratio between peripheral oxygen saturation (SaO2) and FiO2. *P*-values were calculated using a Mann Whitney-U Test for continuous variables and using a Fisher’s Exact test for categorical variables. * BMI of 116 patients was not available. ** Results regarding psychological history are derived from the questionnaire 3 months after discharge. The proportions shown are calculated over a population of 212 patients. *** Results regarding educational level are derived from the questionnaires 1 month after discharge. The proportions shown are calculated over a population of 252 patients. **** Scored the day of first SARS-CoV-2 suspicion. Non-invasive ventilation was defined as use of CPAP or BIPAP; Use of high flow nasal cannula was not included | | | | | | | | | | | |  |
