# Supplementary material for: Psychological distress and health-related quality of life in patients after hospitalization during the COVID-19 pandemic: A single-center, observational study
Source: PLoS One. 2021 Aug 11;16(8):e0255774. doi: 10.1371/journal.pone.0255774 (PMC8357130; doi:10.1371/journal.pone.0255774)
Supplement: S9 Table — (DOCX) [file pone.0255774.s009.docx]

| **S9 Table.** Predictors for the development of probable PTSD, anxiety, and depression, and the self-perceived health state. | | | | | | | | | | | | | | |
| --- | --- | --- | --- | --- | --- | --- | --- | --- | --- | --- | --- | --- | --- | --- |
|  |  | **Probable PTSD** | |  | **Probable anxiety** | |  | **Probable depression** | |  | **Perceived health state** | | | |
|  |  | *Univariate* | |  | *Univariate* | |  | *Univariate* | |  | *Univariate* | | *Multivariate* | |
|  |  | *OR (95% range)* | *P* |  | *OR (95% range)* | *P* |  | *OR (95% range)* | *P* |  | *Beta* | *P* | *Beta* | *P* |
| Time  *(3 months)* |  | 0.12 (0.01-1.10) | **0.06** |  | 0.20 (0.06-0.69) | **0.01** |  | 0.25 (0.08-0.77) | **0.02** |  | 4.4 (1.3-7.6) | **<0.01** | 3.4 (0.2 - 6.6) | **0.04** |
| Age, *years* |  | 0.99 (0.90-1.08) | 0.83 |  | 0.98 (0.92-1.05) | 0.58 |  | 1.03 (0.96-1.10) | 0.47 |  | -0.2 (-0.4- -0.02) | **0.03** | -0.07 (-0.3 - 0.1) | 0.45 |
| Gender  (*female*) |  | 1.15 (0.09-14.6) | 0.92 |  | 1.85 (0.33-10.3) | 0.48 |  | 1.69 (0.31-9.07) | 0.54 |  | -6.9 (-11.7 - -2.2) | **<0.01** | -4.6 (-9.1 - -0.2) | **0.04** |
| ­­Ethnicity  *(Non-Caucasian)* |  | 4.10 (0.35-42.0) | 0.26 |  | 2.86 (0.49-16.9) | 0.25 |  | 1.66 (0.29-9.56) | 0.57 |  | 1.4 (-3.7 - 6.5) | 0.59 |  |  |
| Educational level | | |  |  |  |  |  |  |  |  |  |  |  |  |
| (*High school*) |  | 1.15 (0.02-72.4) | 0.95 |  | 0.77 (0.05-11.0) | 0.85 |  | 1.01 (0.07-14.8) | 0.995 |  | -2.0 (-10.1 - 6.1) | 0.63 |  |  |
| (*Vocational)* |  | 1.19 (0.03-44.9) | 0.93 |  | 0.84 (0.08-8.88) | 0.88 |  | 0.70 (0.06-7.59) | 0.77 |  | -0.2 (-7.3 - 6.9) | 0.96 |  |  |
| (*Higher professional)* |  | 0.33 (0.00-94.4) | 0.70 |  | 0.12 (0.00-4.74) | 0.26 |  | 0.31 (0.02-6.36) | 0.45 |  | 5.1 (-3.3 - 13.6) | 0.23 |  |  |
| (*University*) |  | 0.25 (0.00-235) | 0.70 |  | 0.17 (0.00-7.4) | 0.36 |  | 0.23 (0.01-7.93) | 0.42 |  | 4.9 (-4.3 - 14.2) | 0.30 |  |  |
| Employed (*Yes*) |  | 0.60 (0.03-10.7) | 0.73 |  | 0.49 (0.08-3.1) | 0.45 |  | 0.39 (0.06-2.41) | 0.31 |  | 7.5 (2.6 - 12.4) | **<0.01** | 3.6 (-1.2 - 8.5) | 0.15 |
| Healthcare worker (*Yes*) |  | 2.66 (0.04-187) | 0.653 |  | 1.01 (0.03-31.7) | 0.99 |  | 1.30 (0.04-38.9) | 0.88 |  | 2.1 (-7.7 - 11.9) | 0.68 |  |  |
| Hospital LOS, days |  | 1.02 (0.94-1.11) | 0.57 |  | 0.98 (0.91-1.06) | 0.68 |  | 0.98 (0.91-1.05) | 0.57 |  | -0.1 (-0.3 - 0.1) | 0.36 |  |  |
| Mechanical ventilation (*Yes*) |  | 2.07 (0.09-46.9) | 0.65 |  | 0.61 (0.04-9.46) | 0.73 |  | 0.18 (0.01-5.38) | 0.33 |  | -0.5 (-7.4 - 6.5) | 0.90 |  |  |
| SOFA admission score |  | 1.02 (0.48-2.16) | 0.95 |  | 0.91 (0.53-1.55) | 0.73 |  | 1.02 (0.63-1.66) | 0.94 |  | -0.1 (-1.5 - 1.3) | 0.84 |  |  |
| COVID-19 (*Yes*) |  | 1.11 (0.10-13.1) | 0.93 |  | 0.49 (0.09-2.69) | 0.42 |  | 0.46 (0.09-2.36) | 0.35 |  | 7.0 (2.4 - 11.5) | **<0.01** | 2.7 (-7.1 - 1.7) | 0.23 |
| ICU admission (*Yes*) |  | 1.55 (0.07-33.9) | 0.78 |  | 0.51 (0.04-7.12) | 0.62 |  | 0.14 (0.01-3.90) | 0.25 |  | -0.3 (-6.2 - 6.8) | 0.92 |  |  |
| PTSD severity at 1 month |  | N/A | N/A |  | N/A | N/A |  |  |  |  | -0.5 (-0.7 - -0.3) | **<0.01** | -0.04 (-0.2 - 0.2) | 0.74 |
| Anxiety severity at 1 month |  | N/A | N/A |  | N/A | N/A |  |  |  |  | -2.2 (-2.6 - -1.7) | **<0.01** | -1.1 (-1.9 - -0.3) | **<0.01** |
| Depression severity at 1 month |  | N/A | N/A |  | N/A | N/A |  |  |  |  | -2.2 (-2.6 - -1.8) | **<0.01** | -1.1 (-1.8 - -0.4) | **<0.01** |
| Univariate analysis was performed using mixed logistic (PTSD, anxiety, depression) and linear (perceived health state) models, with the variable of interest as independent variable, and a random intercept for each participant. Each variable with a p-value <0.10 in the univariate mixed model was implemented as independent variable in the multivariate mixed effects regression model. Variables with a p-value <0.05 in the multivariate mixed model were identified as independent predictors. | | | | | | | | | | | | | | |
