# Supplementary material for: Psychological distress and health-related quality of life in patients after hospitalization during the COVID-19 pandemic: A single-center, observational study
Source: PLoS One. 2021 Aug 11;16(8):e0255774. doi: 10.1371/journal.pone.0255774 (PMC8357130; doi:10.1371/journal.pone.0255774)
Supplement: S1 File — (DOCX) [file pone.0255774.s010.docx]

## Supplement 1. Registered study protocol

**1.1 Administrative information**

**TITLE:**  Outcomes after COVID-19

**TITLE:** Psychological morbidity and health-related quality of life in patients hospitalized during the OCVID-19 pandemic; a single- center observational study

**TRIAL REGISTRATION:** Netherlands Trial Register; NL8882

<https://www.trialregister.nl/trial/8882>

**PROTOCOL VERSION** Version 1, April 2020

**ROLES AND RESPONSIBILITIES E-J. Wils, M.D., Ph.D.**

Coordinating Investigator / Project Leader

Department of Intensive Care

Franciscus Gasthuis & Vlietland Group

Kleiweg 500, 3045 PM, Rotterdam, the Netherlands

**J.H. Vlake, B.Sc.**

Principle Investigator

Department of Intensive Care

Franciscus Gasthuis & Vlietland group

Erasmus Medical Center

Kleiweg 500, 3045 PM, Rotterdam the Netherlands.

**M.E. van Genderen, M.D., Ph.D.**

Collaborating Investigator

Department of Intensive Care

Erasmus MC, Rotterdam, the Netherlands

Dr. Molewaterplein 40, 3015 GD, Rotterdam, the Netherlands

**J. van Bommel, M.D., Ph.D.**

Collaborating Investigator

Department of Intensive Care

Erasmus MC, Rotterdam, the Netherlands

Dr. Molewaterplein 40, 3015 GD, Rotterdam, the Netherlands

**SPONSOR**  Franciscus Gasthuis & Vlietland Group

Kleiweg 500

3045 PM, Rotterdam, the Netherlands

**1.2 Introduction**

In December 2019, a new coronavirus, SARS-CoV-2, caused an international outbreak of the respiratory disease COVID-19. The virus rapidly spread around the world, with nowadays cases in Asia, Europa, the United States, and Africa. The spectrum of COVID-19 ranges from mild, self-limiting respiratory tract illness to severe progressive pneumonia, ARDS, pulmonary oedema, multi-organ failure and eventually death.^1-4^ This has led to a tremendous increase of hospital admissions all over the world.

It is known that survivors of critical illness can develop several physical, cognitive and psychological impairments due to their treatment on the Intensive Care Unit (ICU), referred to as the post-intensive care syndrome (PICS), negatively affecting the health-related quality of life and the ability to return to work (HRQoL).^5-8^ In addition, patients admitted to the hospital after unintentional injury, cardiac disease and traumatic brain injury are also at risk for developing posttraumatic stress disorder (PTSD), anxiety and depression, also negatively impacting the HRQoL and ability to return to work.^9-17^ The SARS-CoV-2 outbreak came unexpected, with many patients admitted to the hospital in exceptional conditions. It can therefore be expected that patients admitted to the hospital during the COVID-19 pandemic are at risk for developing psychological impairments, such as PTSD, anxiety and depression, and a decreased functional ability, quality of life and ability to return to their former life.

In the current study, we therefore aim to examine the psychological burden in patients admitted to the hospital with symptoms suggestive of COVID-19 and examine the HRQoL.

**1.2.1 Objectives**

The primary objective is to quantify the psychological burden and the effect on health-related quality of life (HRQoL) of hospitalization during the pandemic, in terms of anxiety, depression and post-traumatic stress disorder, 1 month, 3 months and 12 months after discharge. Secondary objectives are 1) to examine possible differences between COVID-19 and non-COVID-19 patients and 2) to examine possible differences between COVID-19 ICU patients and COVID-19 non-ICU patients.

**1.2.2. Trial Design**

This study is designed as a single center, observational, prospective cohort study.

**1.3 Methods: participants, measures, and outcomes**

**1.3.1 Study setting**

This study will be carried out in the Franciscus Gasthuis & Vlietland hospital, a hospital providing secondary care in Rotterdam, the Netherlands between April 2020 and July 2021.

**1.3.2 Eligibility criteria**

Adults (≥ 18 years old) admitted to the Franciscus Gasthuis & Vlietland hospital between 16th of March 2020 and 28th of April 2020, with symptoms suggestive of COVID-19 and fluent in the Dutch language were eligible for inclusion. Symptoms suggestive of COVID-19 is defined as the presence of respiratory symptoms (e.g. dyspnea, coughing, sore throat, rhinorrhea, saturation <94% or respiratory rate >24/minute), and/or gastro-intestinal symptoms (e.g. diarrhea or vomiting), for a duration of more than 24 hours. Patients had to be subsequently tested for SARS-CoV2 with PCR.

**1.3.3 Exclusion criteria**

Patients were excluded when they already participated in other interventional studies or when their formal house address or e-mail address was unknown.

**1.3.4 Participants timeline**

All patients admitted to the hospital with a suspicion of COVID-19 are registered in the International Severe Acute Respiratory and emergency Infection Consortium (ISARIC, Oxford, United Kingdom) and Castor EDC (Castor EDC, Amsterdam, the Netherlands). Eligible patients are approached 1 month after discharge from the hospital by sending an information letter explaining the nature and aim of the study accompanied by the first set of questionnaires. Participation in the questionnaire takes approximately 15 minutes. Since participation requires only a limited amount of effort and time, the standardized informant-consent procedure was simplified. At the beginning of the questionnaire, patients are asked for consent to use the data gathered using the questionnaires. Patients who agree to participate receive the second set of questionnaires 3 months and 12 months after hospital discharge by e-mail or postal mail, depending on the stated preference. Patients who do not want to participate are asked to return the questionnaire empty. All non-responders are called two weeks after sending the questionnaire as a reminder. Non-responders unable to be reached by telephone are send the second and third questionnaire. Patients who deny participation either via telephone or by returning an empty questionnaire, are excluded from further follow-up.

Data gathered using the questionnaires will be anonymized and will not have an influence on the standard hospital care of these patients in any way possible.

**1.3.5 Sample size**

Due to the nature of the study design and the lack of previous data regarding psychological outcome after COVID-19, we were unable to calculate an a prior sample size.

**1.4 Methods: data collection, management, analysis**

**1.4.1 Data collection Methods**

All data will be collected using electronic data capture system Castor EDC (Castor EDC, Amsterdam, the Netherlands) and International Severe Acute Respiratory and emergency Infection Consortium (ISARIC, Oxford, United Kingdom).

Demographic and treatment related characteristics will be retrieved from patients records and include age, gender, ethnicity, BMI, employment as healthcare worker, medical history, hospital length of stay, ICU admission, ICU length of stay, diagnosis COVID-19; SOFA score, P/F ratio and S/F ratio at first day of COVID-19 suspicion; need and duration of oxygen therapy, non-invasive ventilation, invasive ventilation and prone positioning and insertion of tracheostomy. Educational level, employment status and the psychiatric history will be additionally assessed using a self-composed questionnaire.

Symptoms of PTSD are assessed using the Impact of Event Scale – Revised (IES-R). The IES-R is a validated 22-item questionnaire and assesses subjective distress caused by a traumatic event. The IES-R yields a total score (ranging from 0 to 88, higher scores indicating more severe symptoms) and subscale scores can be calculated for symptoms of intrusion, avoidance, and hyperarousal. A total IES-r score above 24 is classified as clinically significant symptoms of PTSD.^18,19^

Symptoms of depression and anxiety are assessed using the Hospital Anxiety and Depression Scale (HADS). The HADS is commonly used to determine the levels of anxiety and depression that a person is experiencing. The HADS is 14-item scale that generated ordinal data. Seven of the items relate to anxiety and seven relate to depression. The HADS yields a depression and anxiety sum score, ranging from 0 to 21 with higher scores indicating more severe symptoms. A sum score above 8 of either the depression or anxiety subscale is classified as clinically significant symptoms of depression or anxiety.^20,21^

HRQoL is assessed using the European Quality of Life 5-dimensions-5-levels (EQ-5D-5L/EQ-5D) questionnaire and the RAND-36. The EQ-5D measures the HRQoL on five dimensions (mobility, self-care, usual activities, pain/discomfort and anxiety/depression), by which the weight of a health state can be computed, ranging from -0.446 (worst quality of life) to 1.000 (best quality of life). Additionally, patients score their current subjective health state on a visual analogue scale (the EQ-VAS), ranging from 0 (worst health imaginable) to 100 (best health imaginable).^22,23^ The RAND-36 is 36-item, patient reported survey of patient’s health. The RAND-36 yields eight scaled scores, which are weighted sums of the questions in their section. Each scale is directly transformed to a 0-100 scale on the assumption that each question carries equal weight. The scales are vitality, physical functioning, bodily pain, general health perceptions, physical role functioning, social role functioning and mental health.^24,25^

**1.4.2 Data Management**

All data collected during the study will be entered digitally in the electronic data capture system Castor EDC (Castor, Amsterdam, the Netherlands) and the International Severe Acute Respiratory and emergency Infection Consortium (ISARIC, Oxford, United Kingdom). Checks will be applied at the time of data entry into a specific field and/or before the data is written (committed) to the database. Modifications to data written to the database will be documented through the data change system. Data entered into the database will be retrievable for viewing through the data entry applications. All patients are assigned to a unique study identification number and all data will be analyzed anonymously. Original study forms, such as filled in questionnaire, will be stored at each study site in a secured location and will be kept for a minimum of 15 years.

**1.4.3 Statistical Methods**

Baseline demographics will be communicated using descriptive statistics. Continuous variables will be presented as mean (SD) when normally distributed and as median (IQR) when not normally distributed. Categorical variables will be presented as absolute number and relative frequency.

Patients will be stratified based on the confirmation of the diagnosis COVID-19 and, within the COVID-19 patients, on necessity for ICU treatment. Differences between stratifications will be analyzed using a Standard Student’s T-test if the residuals are normally distributed or using a Mann-Whitney U test when the residuals are not normally distributed. Differences in categorical variables will be analyzed using a Chi-squared test or, in case of small sample sizes, a Fisher’s exact test.

Missing data will not be imputed due to the observational nature of the study.

All analyses will be performed using SPSS (version 24.0; SPSS Inc., Chicago, IL) and R for Statistics (R Foundation for Statistical Computing, Vienna, Austria, 2015). A P-value ≤0.05 will be considered statistically significant.

**1.5 Methods: Monitoring**

**1.5.1. Data Monitoring**

No data safety monitoring was needed for this study

**1.5.2. Risk for participants**

No risk applies when patients participate in this study. Data will be processed and analyzed anonymously and will not be retraceable to the individual patient.

**1.6 Ethics and dissemination**

**1.6.1 Research Ethics Approval**

This protocol was reviewed and approved by the Local Institutional Review Boards of the participating study site. The need for ethics approval by an accredited Medical Ethics Committee and written informed consent was waived due to the observational nature of the study.

**1.6.2 Protocol amendments**

Modifications to the protocol which impacted on the conduct of the study, potential benefit of the patient or affected patient safety, including changes of study objectives, study design, patient population, sample sizes, study procedures, or significant administrative aspects were formal amendments to the protocol. Such amendment will be approved by the Institutional Review board prior to implementation and notified to the health authorities in accordance with local regulations.

**1.6.3 Consent or Assent**

The need for written informed consent was waived by the Local Institutional Review Board Informed consent for the use of data gathered using the questionnaires will nevertheless be asked in the first questionnaire. Patients consent by returning a filled-out questionnaire or opt-out by returning an empty questionnaire.

**1.6.4 Confidentiality**

All study-related information will be stored securely at the study site. Returned questionnaire will be stored in locked file cabinets in areas with limited access. All reports, data collection, process, and administrative forms were identified by a coded ID [identification] number only to maintain participant confidentiality. All local databases were secured with password-protected access systems. Forms, lists, logbooks, and any other listings that link participant ID numbers to other identifying information were stored in a separate, locked file in an area with limited access.

Participants’ study information was not be released outside of the study without the written permission of the participant.

**1.6.5 Declaration of interests**

The authors of the protocol have no competing interests.

**1.6.6 Access to data**

All collaborating investigators had full access to view the data concerning their study site during the study. All collaborating investigator had full access to the final data sets. All data was password protected.

**1.7** **References**

1. Wiersinga WJ, Rhodes A, Cheng AC, Peacock SJ, Prescott HC. Pathophysiology, Transmission, Diagnosis, and Treatment of Coronavirus Disease 2019 (COVID-19): A Review. JAMA. 2020.

2. Fu L, Wang B, Yuan T, et al. Clinical characteristics of coronavirus disease 2019 (COVID-19) in China: A systematic review and meta-analysis. J Infect. 2020;80(6):656-665.

3. Guan WJ, Ni ZY, Hu Y, et al. Clinical Characteristics of Coronavirus Disease 2019 in China. N Engl J Med. 2020;382(18):1708-1720.

4. Rothan HA, Byrareddy SN. The epidemiology and pathogenesis of coronavirus disease (COVID-19) outbreak. J Autoimmun. 2020;109:102433.

5. Sukantarat K, Greer S, Brett S, Williamson R. Physical and psychological sequelae of critical illness. Br J Health Psychol. 2007;12(1):65-74.

6. Righy C, Rosa RG, da Silva RTA, et al. Prevalence of post-traumatic stress disorder symptoms in adult critical care survivors: a systematic review and meta-analysis. Critical Care. 2019;23(1):213.

7. Nikayin S, Rabiee A, Hashem MD, et al. Anxiety symptoms in survivors of critical illness: a systematic review and meta-analysis. Gen Hosp Psychiatry. 2016;43:23-29.

8. Rabiee A, Nikayin S, Hashem MD, et al. Depressive Symptoms After Critical Illness: A Systematic Review and Meta-Analysis. Crit Care Med. 2016;44(9):1744-1753.

9. Sheldrick R, Tarrier N, Berry E, Kincey J. Post-traumatic stress disorder and illness perceptions over time following myocardial infarction and subarachnoid haemorrhage. Br J Health Psychol. 2006;11(Pt 3):387-400.

10. Buckland SA, Pozehl B, Yates B. Depressive Symptoms in Women With Coronary Heart Disease: A Systematic Review of the Longitudinal Literature. J Cardiovasc Nurs. 2019;34(1):52-59.

11. Kendrick D, Dhiman P, Kellezi B, et al. Psychological morbidity and return to work after injury: multicentre cohort study. Br J Gen Pract. 2017;67(661):e555-e564.

12. Paredes Molina CS, Berry S, Nielsen A, Winfield R. PTSD in civilian populations after hospitalization following traumatic injury: A comprehensive review. Am J Surg. 2018;216(4):745-753.

13. Van Seben R, Covinsky KE, Reichardt LA, et al. Insight Into the Posthospital Syndrome: A 3-Month Longitudinal Follow up on Geriatric Syndromes and Their Association With Functional Decline, Readmission, and Mortality. J Gerontol A Biol Sci Med Sci. 2020;75(7):1403-1410.

14. Visser E, Gosens T, Den Oudsten BL, De Vries J. The course, prediction, and treatment of acute and posttraumatic stress in trauma patients: A systematic review. J Trauma Acute Care Surg. 2017;82(6):1158- 1183.

15. Walker J, Burke K, Wanat M, et al. The prevalence of depression in general hospital inpatients: a systematic review and meta-analysis of interview-based studies. Psychol Med. 2018;48(14):2285-2298.

16. Kendrick D, Baker R, Hill T, et al. Early risk factors for depression, anxiety and post-traumatic distress after hospital admission for unintentional injury: Multicentre cohort study. J Psychosom Res. 2018;112:15-24.

17. Kendrick D, Dhiman P, Kellezi B, et al. Psychological morbidity and return to work after injury: multicentre cohort study. Br J Gen Pract. 2017;67(661):e555-e564.

18. Creamer M BR, Failla S. Psychometric properties of the impact of event scale—revised. Behav Res Ther. 2003;41(12):1489-1496.

19. Donna McCabe D, APRN-BC, GNP, New York University Rory Meyers College of Nursing. The Impact of Event Scale - Revised (IES-R). Try this, Best Practices in Nursing Care to Older Adults, general assessment series Web site. Published 2019. Accessed 19.

20. Laurin CA, Hottenga JJ, Willemsen G, Boomsma DI, Lubke GH. Genetic analyses benefit from using less heterogeneous phenotypes: an illustration with the hospital anxiety and depression scale (HADS). Genet Epidemiol. 2015;39(4):317-324.

21. Zigmond AS SR. The hospital anxiety and depression scale. Acta Psychiatr Scand. 1983;67(6):361- 370.

22. EuroQol GT. EuroQol-a new facility for the measurement of health-related quality of life. 1990;16(3):199- 208.

23. Versteegh MM VK, Evers SMAA, de Wit GA, Prenger R, Stolk EA. Dutch tariff for the five-level version of EQ-5D. Value health. 2016;19(4):343-352.

24. Hays RD, Sherbourne CD, Mazel RM. The RAND 36-Item Health Survey 1.0. Health Econ. 1993;2(3):217- 227.

25. Care RH. RAND health care, 36-item short form survey (SF-36) scoring instructions. Published 2020. Accessed May 26th, 2020.
