# Supplementary material for: Psychological distress and health-related quality of life in patients after hospitalization during the COVID-19 pandemic: A single-center, observational study
Source: PLoS One. 2021 Aug 11;16(8):e0255774. doi: 10.1371/journal.pone.0255774 (PMC8357130; doi:10.1371/journal.pone.0255774)
Supplement: Attachment — Submitted filename: Review Plosone januari 2021.docx [file pone.0255774.s013.docx]

Review:

This study concerns is a very interesting topic, which has not yet received much attention in research yet. There are, however, some major points I feel should be addressed to make it ready for publication.

Major points:

1. The introduction is very brief, and fails to adequately emphasize the relevance of the study: what does it add to what is known, why is it important to study this.
2. More importantly, no explanation is given for assumed relationships. Please provide theoretical framework for the described relationships. For instance: why do medical problems that require hospitalization lead to psychological problems? What reasons do you have to believe the relationship is causal? The most striking example of this is the hypothesis formulated on page 3. Absolutely no theoretical (nor empirical) foundation for this is given. Why do you expect this? What is the mechanism behind this?
3. The article generally seems to lack focus. Please explain in your introduction why the (primary and secondary) outcomes are important to study during the COVID pandemic. Do you expect this to defer from regular outcomes (outside pandemics) and for all patients admitted with covid-like symptoms or just for the confirmed covid cases? Why? Elaborate. HRQOL and work-resumption suddenly appear when you describe the aims of the study but not before (work-resumption is also completely ignored in the abstract). Why study these?
4. The study has no pre-exposure measurement. The degree to which participants suffered from some degree of psychological problems before hospitalization As a result whether psychological symptoms are caused by hospitalization during the covid-19 pandemic is unknown. There are even studies that suggest that having pre-existing psychological disorders can be a risk factor for contracting COVID-19 (see <https://www.thelancet.com/journals/lanpsy/article/PIIS2215-0366(20)30462-4/fulltext#:~:text=A%20meta%2Danalysis%20of%20pooled,%2C%20poor%20memory%2C%20and%20insomnia)>.
5. No proper distinction is made between posttraumatic stress SYMPTOMS and posttraumatic stress DISORDER. The IES-r is not able to make a diagnosis. It is therefore common practice to speak of (high levels of) PTSS, or probable PTSD. Furthermore, if you really wanted to examine predictors of PTSD, not PTSS a linear regression is not the correct analysis, after all, one either has PTSD or not.
6. Relate outcome levels to outcome levels outside pandemic conditions in previous studies. Can we conclude that there is a problem specific to pandemics?
7. Educational level is added to the regression models as a linear variable instead of dummy variables. This is a categorical variable with unequal distances between categories. The resulting coefficient is therefore meaningless.
8. The description of the measured used in the study is not sufficient. Please add range of scales and reliability statistics within your sample. Also elaborate on operationalization of other variables such as demographics. For instance, how is ethnicity operationalized? If more than two categories are used in the regression (such as native and non-native) see my previous comment as well.
9. No distinction is made in the regression analyses between the subgroups. Theoretically it would be interesting to distinguish the COVID and non-COVID patients. Ideally this would be done using a multilevel model to determine the impact of group membership on the influence of predictors, but the samples are probable too small for this.
10. I think the statement on p16 that “Collectively, this suggest that participating patients are grossly representative for the total cohort” is too bold. Your response rate is quite low, and examining patient records do not inform you how they would have scored on the outcome measures. A nice idea to ask a small sample of non-responders to respond anyway, but self-selection here is of course also a factor. You could consider comparing outcomes between the full responders (T1 and T2) and those that responded at T1 only.

Minor points:

1. I prefer a table where all primary and secondary outcomes can be seen, instead of a series of boxplots (which seem rather archaic). Having to look up a long list of supplementary tables is not helping your readers understand your work. especially as the tables put in the supplementary section are core study results. Readers are now forced to resort to the long summary of the results in the text, which do not give all information. I propose to put them into the article, and to merge them, and move the box-and-whisker plots to the supplementary section for the fans. This also means shortening the results section as not all results in the table then need to be described in text.
2. The Venn-diagrams look nice, but also add little. Please put these in the supplementary section as well.
3. There is no table for your regression analyses, not even in the supplements! Please add these (to the article, not the supplementary section).
4. Please check for spelling and grammar errors such as “Predictors off psychological outcomes” on page 12. Or also see comment 10 these suggest/this suggests.
5. When describing outcomes (especially when tables are not included) please add direction of association. E.g.: does having had a job before hospitalization lead to higher or lower psych symptoms?
6. Page 3: “the pandemic peak”. Is no longer applicable as there are 2 peaks at this point in time.
7. Provide a benchmark for HRQOL. You state it is low, but what are reference levels for for instance the general population?
