# Supplementary material for: Psychological distress and health-related quality of life in patients after hospitalization during the COVID-19 pandemic: A single-center, observational study
Source: PLoS One. 2021 Aug 11;16(8):e0255774. doi: 10.1371/journal.pone.0255774 (PMC8357130; doi:10.1371/journal.pone.0255774)
Supplement: Attachment — Submitted filename: Comments to authors.docx [file pone.0255774.s014.docx]

**Comments to the authors;**

**“Psychological distress and health-related quality of life in patients after hospitalization during the COVID-19 pandemic: a single-centre, observational study”**

The study topic is relevant and interesting but is not clearly reported.

**Introduction**

1. The introduction is too shallow. On top of what is written, the methods of psychological distress measurements used in this study need to be briefly described (i.e PTSD, depression, and anxiety measures using the Impact of Event Scale – Revised (IES-R) and the Hospital Anxiety and Depression Scale (HADS)). Briefly discuss as well how to measure health related quality of life using EQ-5D and RAND-36. This brief description should be done in a very concise and understandable manner.

2. The problem statement of this study is not clearly stated: i.e “ Data from the previous SARS and MERS epidemics support this concern and more recent studies suggest that up to 50% of COVID-19 patients suffers from psychological distress up to one month after hospital discharge”. Looking at the results of this study (i.e of one of the results; “patients admitted for other illnesses more suffered psychologically than COVID-19” patients) , I was expecting to read a statement contradicting this to support your study results.

3. The title, the aim of the study and results do not link well. Authors also say “Additionally, we tested the hypothesis that the psychological distress was higher in COVID-19 confirmed patients, and in those treated in ICUs”. Where is this hypothesis? Why the authors talk about an hypothesis which is not stated in the study?

**Materials and methods**

1. “**Study design and setting**”; when I read this heading, I expect to get information on study design and setting only. More information given here is about ethical consideration. For “study setting”, one should expect to read more of the description of the location, department, environment, infrastructure, etc… of the study place. The authors can either name this heading “**study design and ethical consideration**” or modify the content to suite the heading, and shift the content to an ethical heading. **NB: Please read more on study setting.**

**2.“participants”;** in this section, the inclusion and exclusion criteria are not clear. “the presence of respiratory symptoms (e.g., dyspnoea, coughing, sore throat, rhinorrhoea, saturation 24/minute) and/or gastro-intestinal symptoms (e.g., diarrhoea or vomiting) with a duration ≥24 hours”. The study inclusion and exclusion should be clear. The authors should not give examples of symptoms but should clearly state which symptoms constituted inclusion criteria and which constitute exclusion criteria. I suggest a heading “**Inclusion and exclusion criteria**” **or Eligibility criteria**.

**3. “Procedures”;** With this heading two important considerations come up: (1) Data collection tools/instruments; after reading this study, 4 questionnaires and one data collection tool are supposed to be used yet none is described in this section. Please discuss briefly these tools;”(Impact 93 of Event Scale – Revised (IES-R), the Hospital Anxiety and Depression Scale (HADS), EQ-5D, RAND 36 and demographic data collection tool”

Authors say “Patients who were unable to understand the Dutch language or did neither have a formal home address nor e-mail address were excluded”. The dutch language is another issue on data collection tool used to collect data. It should come clearly what was the language of the questionnaires. “did the authors used a standard questionnaire in Dutch”? If yes, clarify how this was translated. If the original questionnaire was in English, clarify how it was translated in Dutch then back in English for data entry.

(2) **Sampling procedure**; it is not clear how the sampling procedure was done. Random sampling? Convenient sampling?.....The author needs to explain how the sampling procedure was done and clarify the sample size as well.

(3) It is also not clear who collected the data. Were authors collected data themselves? or trained research assistants to collect data.

**4.”Outcomes”;** In this section, it is expected to explain how authors have reached the mentioned outcomes. I.e for EQ-5D questionnaire, the author must clarify which **published algorithm** used to allow **a CROSSWALK** to measure utility. “ The questionnaire should score using a **published algorithm** **based on community survey in the country or neighboring country using standard Gamble, Time trade off or visual analogue scale(VAS)”.**

**5.”Statistical analysis”;** The analysis of this study is well done but poorly reported. i.e firstly, the author reported “ correlations between psychological outcomes and baseline/treatment characteristics were analyzed using a Spearman’s rho”. The authors should report the correlation coefficients. Secondly, the authors said “Possible predictors for psychological outcomes were analyzed using a **multivariate linear regression model.** Correlations with a p-value below 0.10 were included in a **multivariate linear regression model** to identify the predictor”. Please review this statement if this is what you wanted to say and rectify.

**Results**

1.In the participants section, the results are not clearly explained. It is hard to understand this paragraph; “A total 123 of 282 individuals participated (45%; Fig 1); 252 at one month (40%) and 201 of 365 (55%) at three months post discharge. Non-participating patients either declined participation (n=262) or did not respond (n=79)”. It is also difficult to understand the response rate of the 4 questionnaires distributed twice (at 1 month and 3 months). Please restructure this section by shading more light to this section.

2. It is also not clear when the data collection was done. Please clarify these i.e; in the Study design section; the authors say “This single-centre, observational cohort study was conducted in the Franciscus Gasthuis & Vlietland hospital in Rotterdam, the Netherlands, from March 16 to September 14, 2020”. And in Participants section; the authors say “Eligible patients were aged ≥18 years and hospitalized between March 16 and April 28, 2020”. And in results section, the authors say as well “From March 16 to April 28, 2020, 623 eligible patients were admitted with symptoms suggestive of COVID-19”.

**3. My Suggestion on how the results should be presented in results section**

a. Introduce the results section and present demographic characteristics in a table, comment your results from the table

b. Present psychological distress results in a table or a clear figure, then comment the table or figure in an understandable manner. Show and comment on any P-value

c. Present the health related quality of life in a table or a figure, comment the results. Show and comments P-values if any

d. Present the predictors of psychological outcomes and quality of life in a table showing the regression results and P-values, correlation coefficients and comment the table.

**Discussion and references;** I cannot comment on discussion and references for now, if results are not presented in an understandable manner
