# Supplementary material for: Psychological distress and health-related quality of life in patients after hospitalization during the COVID-19 pandemic: A single-center, observational study
Source: PLoS One. 2021 Aug 11;16(8):e0255774. doi: 10.1371/journal.pone.0255774 (PMC8357130; doi:10.1371/journal.pone.0255774)
Supplement: Attachment — Submitted filename: Response to the reviewers.docx [file pone.0255774.s015.docx]

**Response to the handling editor:**

Dear Dr. Vlake,

Thank you for submitting your manuscript to PLOS ONE. After careful consideration, we feel that it has merit but does not fully meet PLOS ONE’s publication criteria as it currently stands. Therefore, we invite you to submit a revised version of the manuscript that addresses the points raised during the review process.

I have read your interesting manuscript with pleasure and received the comments of three independent reviewers. The critical, detailed, very clear and helpful comments of the reviewers are included in this letter. I fully agree and share their comments and will try not repeat them in my comments below.

**R:** Dear Dr. Peter G. van der Velden,

We really appreciate that you are handling our manuscript, and that you are willing to give us the opportunity to thoroughly revise our manuscript. The suggestions, concerns, and comments raised gave us the opportunity to further improve our manuscript, and we have made point-by-point responses to all comments below.

For the referral to certain pages or lines within the manuscript; all referrals are based on the submitted marked-up manuscript, in which the changes made during the revision are highlighted.

1) My general impression is that you have so many study variables you all want to use (among a relatively small group), that the condensed format you have chosen to report your findings in, hinders (among other things) the readability of your manuscript. I would like to suggest to rethink your analyses: your main aim seems to be the assessment of the course of PTSD, anxiety and depression symptom, and quality of life among different patient groups over two waves. One major problem you face is the non-response at T1 or T2 which hinders clarity about your study sample (and N). Using mixed effects models could be a way to solve this problem (or using multiple imputation). Of course, you can enter relevant covariates in your analyses. Perhaps covid and non-covid groups could be merged to limit the number of groups/comparisons (with IC as dummy variable). Your second aim seems to be the assessment of predictors of your dependent variables. As also mentioned by the reviewers, this element in your manuscript lacks clarity and focus. It seems more data driven than anything else (see long list of correlations). I would like to suggest to limit the number of predictors, and use current knowledge on predictors or PTSD, etc to choose a limit number of relevant predictors. There are several reviews and meta-analyses available that are very helpful in this perspective.

**R:** We understand the comment raised, and in hindsight agree that we shared a too large amount of information within the paper, and that this hindered the readability of the manuscript. The main aim of the study was indeed to assess the course of psychological sequelae (symptoms of PTSD, anxiety, and depression) and quality of life in time after hospital discharge in patients included during the first COVID-19 pandemic wave. To address this comment, we have made the following changes:

1) We adjusted **figure 1** to make it more insightful how inclusion was performed.
2) As suggested by the editor, we have re-analyzed the entire results section using regression models, adjusting for baseline differences. In order to determine possible predictors, we have used linear and logistic mixed models.

We therefore added:

“*Differences in outcome measures between stratifications were analyzed using linear or logistic regression models for continuous and categorical outcomes, respectively, adjusting for baseline differences between groups, in which the ‘stratification’ and at baseline differing characteristics served as independent variables.”* (Methods, page 9, lines 172-174).

“*To identify possible predictors for psychological symptomatology or quality of life, we first conducted a univariate linear or logistic mixed model with possible predictors as independent variables (S1. Table). Variables with a p-value ≤0.10 were added in the multivariate linear or logistic mixed model to determine predictors.*” (Methods, page 9, lines 180-182)

“*Psychological outcomes of randomly selected non-responders were compared with those of responders using a linear or logistic regression model, adjusting for baseline differences between groups, in which the ‘stratification’ and at baseline differing characteristics served as independent variables.*” (Methods, page 9, lines 184-186)

Additionally, we changed the Results section within the Abstract section accordingly:

*“282 of 623 eligible patients participated in this study (median age 64 years, 36% female). 16% and 12% of these patients reported clinically relevant symptoms of PTSD, 29% and 20% clinically relevant symptoms of anxiety, and 32% and 25% clinically relevant symptoms of depression after one and three months, respectively. ICU patients reported less probable depression, but no differences were found in PTSD, anxiety, or overall HRQoL. COVID-19 patients had a worse physical and mental quality of life one month after discharge, and ICU patients reported a better physical quality of life three months after discharge. PTSD severity was predicted by time after discharge and not being Caucasian. Severity of anxiety was predicted by time after discharge, age, educational level, employment status, COVID-19 diagnosis, and not being Caucasian. Depression severity was predicted by time after discharge and educational level. 49% and 76% of previously working patients had resumed work, and patients worked a median of 30 hours less at both follow-up time-points. COVID-19 patients more frequently experienced a financial decline. ”* (Abstract, page 2-3, lines 40-49)

3) We limited the number of variables included for the prediction model, and these variables were selected based on existing literature on risk factors for developing psychological sequelae after hospitalization. We therefore added the following sentences into the methods section:

*“These predictors were chosen based on previous literature, and included age, gender, ethnicity, educational level, duration of admission, ICU admission, COVID-19 diagnosis and work before admission, and severity of disease in terms of SOFA score at the first day of COVID-19 suspicion (day of enrolment). Literature supporting these predictors is depicted in S1. Table in the Supporting Information.*” (Methods, page 8, lines 158-162)

2) Your very short introduction section lacks a proper overview of what is known about the effect is illness/ hospitalization on mental health (including PTSD), and introduction of study variables. This must be added to be able to understand your analyses.

**R**: We agree with the editor that the introduction sections in its present state is limited, and therefore added information about the effect of illness/hospitalization on mental health and the rationale for using certain variables as predictors for psychological distress and quality of life.

Concordantly, we added the following:

“*Symptoms of the infectious disease caused by the SARS-CoV-2 virus, coronavirus disease-19 (COVID-19), vary from mild respiratory and gastro-intestinal symptoms to severe acute respiratory failure, requiring respiratory support in an Intensive Care Unit (ICU) (2-4).*” (Introduction, page 4, lines 64-66)

“*…; symptoms of depression and anxiety occur in 12-67% of hospitalized patients; symptoms of post-traumatic stress disorder (PTSD) in 19%-45% (8-15). Survivors of ICU treatment are known to be at risk for psychological impairments including symptoms of depression, anxiety and PTSD. These symptoms occur in up to 20%-67% of ICU survivors (16-21). Post-hospitalization psychological impairments negatively impact on the ability to return to work and the health-related quality of life (HRQoL) (13, 17, 18, 20).*” (Introduction, page 4, lines 77-81)

“*The risk of psychological impairments is expected to be even higher in patients hospitalized during the COIVD-19 pandemic due to the exceptional circumstances and uncertainties (22, 23).*“ (Introduction, page 4, lines 82-83)

3) According to your manuscript, 623 patients were eligible and 171 participated in both surveys. The non-response should be included in the text before the results section. In general, the manuscript lacks a clear overview in the text and figures about the number of respondents the analyses and figures refer to. This need to be solved.

**R:** We agree that the manuscript in its present state lacks this clear overview. We therefore rewrote the results section, expanded the introduction, and changes the figures and tables in the text, and removed several tables from the supplementary materials to overcome this issue.

With regard to the inclusion rate; we adjusted figure 1 to make it more insightful. We also replaced the paragraph on this matter prior to the ‘Participants’ section within the Results section. In addition, we addressed the non-inclusion rate by adding the following sentence to the result section:

“*Non-participating patient either declined participation (n=262) or did not respond to the questionnaires or to the reminding phone calls (n=79).*” (Results, page 10, lines 195-197)

4) You compared subgroups of patients (see also figures) while at the same time you showed that these subgroups differed at baseline. In your analyses you did not control for these differences which is not allowed. Your figures in figure 3 in which you compare groups must therefore be omitted. The added value of your figures was unclear to me.

**R:** We agree with the editor’s comment that we should control for baseline differences. In line with the response to the editor’s previous comments, we changed the analysis within the results section, and used regression and mixed models instead of non-controlling tests (i.e., Wilcoxon or t-test or T-test).

With regard to the *Figure 3* and *Figure 4*, in line with the comment raised by the editor, we have omitted these figures, and prioritized tables instead.

5) The outcome measures need to be clarified much more in detail (including adding cronbachs alphas for all assessments at the two waves). You did not examine mental disorders using the IESR and HADS. Please define high scores as probable PTSD etc. and avoid the use of the term disorder in this perspective.

**R:** We agree with the editor that the description of the used questionnaires was limited, and, in accordance with the raised concern, we described the outcome measures in more detail.

Therefore we added the following sentences:

“*The IES-R is a validated 22-item questionnaire that quantifies the subjective distress a person is experience after a traumatic event (32). The IES-R yields a total score, ranging from 0-88 (higher scores indicating more severe symptoms), and subscale scores can be calculated for symptoms of intrusion, avoidance and hyperarousal.*” (Methods, page 7, lines 132-135)

“*The HADS is commonly used to determine the levels of anxiety and depression that a person is experiencing (34). The HADS is 14-item scale that generated ordinal data. Seven of the items relate to anxiety and seven relate to depression. The HADS yields a depression and anxiety sum score, ranging from 0 to 21 with higher scores indicating more severe symptoms.*” (Methods, page 7, lines 135-138)

*“The EQ-5D measures the HRQoL on five dimensions (mobility, self-care, usual activities, pain/discomfort and anxiety/depression), by which the weight of a health state can be computed into an EQ-5D Time Trade Off (TTO) score. This score ranges from – 0.446 (worst quality of life) to 1.000 (best quality of life). Additionally, patients score their current subjective health state on a visual analogue scale (the EQ-VAS), ranging from 0 (worst health imaginable) to 100 (best health imaginable) (36, 37).”* (Methods, page 7-8, lines 141-145)

“*The RAND-36 is a 36-item, self-reported survey of HRQoL, and consists nine scales scores, which are the weighted sums of the questions in their section (38). Each scale is directly transformed to a scale ranging from 0 (worse score) to 100 (best score) on the assumption that each question carries an equal weight. The nine scores are; …*” (Methods, page 8, lines 148-150)

“*Resumption to work and financial decline due to the hospitalization were was additionally assessed using self-composed questionnaires.*” (Methods, page 8, lines 154-155)

The editor also rightly point out that we did not determine the presence of psychiatric disorders, but rather the presence of symptomatology, and thus the probability of the disorders. We therefore removed ‘disorders’ from the manuscript, and used probable PTSD, anxiety, and depression instead.

6) You used the Bonferroni correction without explaining why. Without a proper explanation I urge you to drop this often misused correction method. Please consult papers on this issue or Lakens´ blog. He wrote an interesting piece about this correction. <http://daniellakens.blogspot.com/2016/02/why-you-dont-need-to-adjust-you-alpha.html>

**R**: We agree with the editor that the use of the Bonferroni correction was lacking. As we changed our analysis by using regression and mixed models, we did no longer need correction and therefore deleted the following from the manuscript:

“We corrected for multiple testing by using a Bonferroni correction.”

7) Because of all the serious issues raised by the reviewers and my ow comments that need to be addressed first, it is not useful to comment of the discussion section at this stage.

**R:** We understand the editor that reviewing the discussion at this stage was not useful. Of note, we revised our discussion based on the revisions, and hope that our revisions are sufficient to review our discussion section.

8) Please submit your revised manuscript by Mar 13 2021 11:59PM. If you will need more time than this to complete your revisions, please reply to this message or contact the journal office at plosone@plos.org. When you're ready to submit your revision, log on to https://www.editorialmanager.com/pone/ and select the 'Submissions Needing Revision' folder to locate your manuscript file.

**R:** We have submitted the revised manuscript prior to the assigned deadline.

Please include the following items when submitting your revised manuscript:

9) A rebuttal letter that responds to each point raised by the academic editor and reviewer(s). You should upload this letter as a separate file labeled 'Response to Reviewers'.

**R:** The current document responds to the points raised by the academic editor and reviewers.

10) A marked-up copy of your manuscript that highlights changes made to the original version. You should upload this as a separate file labeled 'Revised Manuscript with Track Changes'.

**R**: We submitted a marked-up copy of the manuscript highlighting all chances made to the manuscript.

11) An unmarked version of your revised paper without tracked changes. You should upload this as a separate file labeled 'Manuscript'.

**R**: We have submitted a clean version of the manuscript, without tracked changes, to the revised submission of the manuscript

**Response to the Reviewer's Responses to Questions**

1. Is the manuscript technically sound, and do the data support the conclusions?

The manuscript must describe a technically sound piece of scientific research with data that supports the conclusions. Experiments must have been conducted rigorously, with appropriate controls, replication, and sample sizes. The conclusions must be drawn appropriately based on the data presented.

Reviewer #1: Yes

Reviewer #2: Partly

Reviewer #3: Partly

**R**: We have changed the introduction, methods, and results according to the comments raised by the reviewers, and hope this has resulted in the manuscript being technically sound, and to more clearly illustrate the data supporting the conclusions.

________________________________________

2. Has the statistical analysis been performed appropriately and rigorously?

Reviewer #1: No

Reviewer #2: No

Reviewer #3: Yes

**R**: We have changed the analysis methods by using regression and mixed models. We thereby hope that the statistical analysis has been performed appropriately and rigorously.

________________________________________

3. Have the authors made all data underlying the findings in their manuscript fully available?

The PLOS Data policy requires authors to make all data underlying the findings described in their manuscript fully available without restriction, with rare exception (please refer to the Data Availability Statement in the manuscript PDF file). The data should be provided as part of the manuscript or its supporting information, or deposited to a public repository. For example, in addition to summary statistics, the data points behind means, medians and variance measures should be available. If there are restrictions on publicly sharing data—e.g. participant privacy or use of data from a third party—those must be specified.

Reviewer #1: No

Reviewer #2: Yes

Reviewer #3: No

**R:** We have submitted the de-identified data as supporting information, and thereby made all data underlying the findings in the manuscript fully available.

We added the following to the Data Sharing Statement in the Declarations:

*“The datasets used and/or analyzed during the current study are included in the supporting information files.“* (Page 27, line 485)

________________________________________

4. Is the manuscript presented in an intelligible fashion and written in standard English?

PLOS ONE does not copyedit accepted manuscripts, so the language in submitted articles must be clear, correct, and unambiguous. Any typographical or grammatical errors should be corrected at revision, so please note any specific errors here.

Reviewer #1: Yes

Reviewer #2: Yes

Reviewer #3: No

**R:** We have copy-edited the entire manuscript to ensure it is presented in an intelligible fashion and written in Standard English.

________________________________________

**Response to Comments raised by Reviewer #1:**

The article present san interesting piece of research of psychological reactions and symptoms of hospitalized patients with COVID symptoms. The literature review is clear, and the authors present the methods in an adequate way.

1. However, there are two main issue with the paper that I think might be considered by the authors regard to description of what they are studying and the report of results. The authors declare that they are presenting data for psychological disorders, and not what they are presenting is symptoms reported by patients, more information is needed to arrive to diagnosis, so I suggest changing that presentation of the results.

**R:** We thank you for reviewing our manuscript, and for your useful comments. We agree that we should not have used the term ‘disorder’, a similar concern was raised by the academic editor (comment 5). We therefore discarded ‘disorder’ from the manuscript and changed it with probable PTSD, anxiety, or depression.

2. Moreover, I think the methods section can include more information and justification of the decisions made. It is important that the authors declare why they choose one and three months as appropriate time points to collect the data.

**R:** We agree with the reviewer that the methods section should have included more information. A similar comment was raised by the academic editor (comment 5).

With regard to the chosen time-points to collect the data, we added the following to the manuscript:

“*Psychological outcomes were assessed one month and three months after hospital discharge to 1) compare results with other research concerning psychological outcomes after hospital admission due to COVID-19 or similar coronaviruses; studies up till now mainly reported data of the first month after discharge (27) and 2) to evaluate the course of psychological symptoms and HRQoL in time after discharge.*“ (Methods, page 8, lines 163-167)

3. In the statistical analysis description, authors must include a justification of why to choose variables with correlations with a p-value below 0.10 to be included in a multivariate linear regression model.

**R:** First, based on the comment raised by the academic editor (comment 1), we have changed our analysis using mixed models for the identification of possible predictors. Therefore, we have analyzed all possible predictors using a univariate mixed model and used the variables with a p-value ≤0.10 for the full (multivariate) mixed model. Thereby, we have discarded the use of correlations from original manuscript.

Within prediction models, there are several manners to select variables, of which one it to perform univariate analyses on each variables and include those with a small (≤0.10) p-value into multivariate (mixed) models.

Concordantly, we added the following:

“*Differences in outcome measures between stratifications were analyzed using linear or logistic regression models for continuous and categorical outcomes, respectively, adjusting for baseline differences between groups, in which the ‘stratification’ and at baseline differing characteristics served as independent variables.”* (Methods, page 9, lines 172-174)

*“To identify possible predictors for psychological symptomatology or quality of life, we first conducted a univariate linear or logistic mixed model with possible predictors as independent variables (S1. Table). Variables with a p-value ≤0.10 were added in the multivariate linear or logistic mixed model to determine predictors.”* (Methods, page 9, lines 180-182)

4. Moreover, it is important to justify why they used a multivariate linear regression for this purpose, the other analysis suggest that the data do not follow a parametric distribution.

**R:** We agree with the reviewer that using a multivariate linear regression model was a suboptimal choice. We have changed the analysis within the manuscript, and now used mixed models instead, of which the assumptions rely less on the distribution of the variables within the model. For the changes we made to the statistical analysis, see our response to your previous comment.

5. In the results section, the confusion in the section "Predictors of psychological outcomes and quality of life" remains as a result of the lack of the explanation in the statistical analysis section. If the authors maintain the idea to include de multivariate linear regression, I suggest first to declare, in the paper, which variables will be included in the model, and then to present the results of the significance of the model and betas, SE and p values for all the variables.

**R:** We agree with the reviewer that we did not explain our choice of variable, nor the analysis of predictors, well enough. A similar comment was raised by the academic editor (comment 1).

With regard to the choice of predictive variables, we have added the following sentences into the manuscript:

“*These predictors were chosen based on previous literature, and included age, gender, ethnicity, educational level, duration of admission, ICU admission, COVID-19 diagnosis and work before admission, and severity of disease in terms of SOFA score at the first day of COVID-19 suspicion (day of enrolment). Literature supporting these predictors is depicted in S1. Table in the Supporting Information.*” (Methods, page 8, lines 158-162).

In addition, in the results section, we have added the betas, SE, and p-values of all significant variables used in the models, and added tables depicting the outcomes of the univariate and multivariate mixed models, including betas and p-values, to predict the severity of psychological symptoms and HRQoL in table 4 and 5 in the manuscript, and the tables concerning the predictors of the prevalence of psychological symptoms in table S3.

6. Also, in the results section, in line 125 the authors indicate that 24 of the 201patients reported a history of mental illness. Please clarify why only there is information about this issue of 201 patients of the 282 patients that participate. If they rest of participants did not respond to that question, please state that in the article.

**R:** We agree with the reviewer that this is not well described in the manuscript. Patients were indeed asked about their mental history and could self-determine whether they wanted to share this history with us.

Therefore, we added the following sentence into the manuscript:

“*Patients were asked for their psychiatric history and could choose whether or not to answer.*” (Methods, page 8, line 155-156).

“*…, who were willing to share this information.*” (Results, page 10, line 204).

**Response to Comments raised by Reviewer #2:**

This study concerns a very interesting topic, which has not yet received much attention in research yet. There are, however, some major points I feel should be addressed to make it ready for publication.

Major points:

1. The introduction is very brief, and fails to adequately emphasize the relevance of the study: what does it add to what is known, why is it important to study this.

**R:** We agree with the reviewer that the introduction was to brief, and a similar concern was raised by the academic editor (comment 2). We therefore expanded the introduction, as further described in our response to comment 2 of the academic editor.

2. More importantly, no explanation is given for assumed relationships. Please provide theoretical framework for the described relationships. For instance: why do medical problems that require hospitalization lead to psychological problems? What reasons do you have to believe the relationship is causal? The most striking example of this is the hypothesis formulated on page 3. Absolutely no theoretical (nor empirical) foundation for this is given. Why do you expect this? What is the mechanism behind this?

**R:** We agree with the reviewer that more information should be added to the manuscript on the rationale and choices made within our study.

To the introduction, we added the following:

*“…; symptoms of depression and anxiety occur in 12-67% of hospitalized patients; symptoms of post-traumatic stress disorder (PTSD) in 19%-45% (8-15). Survivors of ICU treatment are known to be at risk for psychological impairments including symptoms of depression, anxiety and PTSD. These symptoms occur in up to 20%-67% of ICU survivors (16-21). Post-hospitalization psychological impairments negatively impact on the ability to return to work and the health-related quality of life (HRQoL) (13, 17, 18, 20).” (Introduction, page 4, lines 77-81)*

*“The risk of psychological impairments is expected to be even higher in patients hospitalized during the COIVD-19 pandemic due to the exceptional circumstances and uncertainties (22, 23).“ (Introduction, page 4, lines 82-83)*

Additionally, we added the following to the methods:

“*These predictors were chosen based on previous literature, and included age, gender, ethnicity, educational level, duration of admission, ICU admission, COVID-19 diagnosis and work before admission, and severity of disease in terms of SOFA score at the first day of COVID-19 suspicion (day of enrolment). Literature supporting these predictors is depicted in S1. Table in the Supporting Information.*” (Methods, page 8, lines 158-162).

3. The article generally seems to lack focus. Please explain in your introduction why the (primary and secondary) outcomes are important to study during the COVID pandemic. Do you expect this to defer from regular outcomes (outside pandemics) and for all patients admitted with covid-like symptoms or just for the confirmed covid cases? Why? Elaborate. HRQOL and work-resumption suddenly appear when you describe the aims of the study but not before (work-resumption is also completely ignored in the abstract). Why study these?

**R:** We thank the reviewer for this comment. First, we added the following to the introduction to emphasize that we hypothesize that patients, as in our cohort, have an increased risk of developing psychological symptomatology:

*“…; symptoms of depression and anxiety occur in 12-67% of hospitalized patients; symptoms of post-traumatic stress disorder (PTSD) in 19%-45% (8-15). Survivors of ICU treatment are known to be at risk for psychological impairments including symptoms of depression, anxiety and PTSD. These symptoms occur in up to 20%-67% of ICU survivors (16-21). ” (Introduction, page 4, lines 77-81)*

*“The risk of psychological impairments is expected to be even higher in patients hospitalized during the COIVD-19 pandemic due to the exceptional circumstances and uncertainties (22, 23).“ (Introduction, page 4, lines 81-82)*

In addition, we have added the following to explain why we also study HRQoL and work resumption:

“*Post-hospitalization psychological impairments negatively impact on the ability to return to work and the health-related quality of life (HRQoL) (13, 17, 18, 20).”* (Introduction, page 4, lines 80-81)

We added the following to the abstract, as this point was indeed missing in the abstract of our original manuscript:

“*Illnesses requiring hospitalization are known to negatively impact psychological well-being and health-related quality of life (HRQoL) after discharge.*” (Abstract, page 2, lines 24-25)

“*…. is expected to be higher due to the exceptional circumstances within and outside the hospital during the pandemic surge* ” (Abstract, page 2, lines 26-27)

“*We also aimed to determine HRQoL and work resumption, to explore predictors for psychological distress and HRQoL, and to examine whether psychological distress was higher in COVID-19 confirmed patients, and in those treated in ICUs.*“ (Abstract, page 2, lines 29-31)

“*Health-related quality of life (HRQoL) and work resumption were secondary outcomes. Predictors for psychological distress and HRQoL were explorative outcomes.*” (Abstract, page 2, lines 36-37)

“*PTSD severity was predicted by time after discharge and not being Caucasian. Severity of anxiety was predicted by time after discharge, age, educational level, employment status, COVID-19 diagnosis, and not being Caucasian. Depression severity was predicted by time after discharge and educational level. 49% and 76% of previously working patients had resumed work, and patients worked a median of 30 hours less at both follow-up time-points. COVID-19 patients more frequently experienced a financial decline.*“ (Abstract, page 2, lines 45-49)

4. The study has no pre-exposure measurement. The degree to which participants suffered from some degree of psychological problems before hospitalization As a result whether psychological symptoms are caused by hospitalization during the covid-19 pandemic is unknown. There are even studies that suggest that having pre-existing psychological disorders can be a risk factor for contracting COVID-19 (see <https://www.thelancet.com/journals/lanpsy/article/PIIS2215-0366(20)30462-4/fulltext#:~:text=A%20meta%2Danalysis%20of%20pooled,%2C%20poor%20memory%2C%20and%20insomnia)>.

**R:** We agree with the reviewer that the lack of a pre-exposure measure is missing. A pre-exposure measure was unfortunately not attainable giving the study circumstances. However, as recently discussed by Geense et al., pre-exposure in unplanned admitted patients (all of the included patients in our cohort), depends on retrospective recollection of psychological functioning and HRQoL. Moreover, pre-exposure PTSD scoring is meaningless, as PTSD is inherently related to the traumatizing event under study. Notwithstanding its shortcomings, we strongly agree with the reviewer that future studies on psychological and HRQoL recovery should strive for pre-exposure measure.

We addressed this comment in the discussion section:

“*First, non-COVID-19 patients suffered from more comorbidities and were older, indicative of a worse pre-existent health status, but baseline psychological status and HRQoL of our cohort were unfortunately not available. We thus cannot formally rule out that the pre-existing psychological well-being was poorer and resilience lower, predisposing for impaired psychological recovery (47).*” (Discussion, page 24, lines 410-413)

Additionally, we added the following to the discussion section to emphasize this limitation;

“*First, the high incidence of psychological symptomatology and the poor HRQoL post-discharge may be either attributed to factors related to hospitalization or to baseline psychological imbalances. Baseline psychological status and HRQoL in our cohort was not available, but only a few patients in our cohort reported a history of mental illness, suggesting that hospitalization rather than pre-existing psychological distress relates to post-discharge symptomatology. To overcome this issue, we believe that future studies on psychological and HRQoL recovery should strive for a best effort to obtain a pre-exposure evaluation (53).*” (Discussion, page 25, lines 439-445)

5. No proper distinction is made between posttraumatic stress SYMPTOMS and posttraumatic stress DISORDER. The IES-r is not able to make a diagnosis. It is therefore common practice to speak of (high levels of) PTSS, or probable PTSD. Furthermore, if you really wanted to examine predictors of PTSD, not PTSS a linear regression is not the correct analysis, after all, one either has PTSD or not.

**R**: We fully agree with the reviewer, and similar concerns were raised by the academic editor and reviewer 2. We have therefore discarded ‘disorder’ from the manuscript, and used probable PTSD, anxiety, and depression.

6. Relate outcome levels to outcome levels outside pandemic conditions in previous studies. Can we conclude that there is a problem specific to pandemics?

**R:** We thank the reviewer for this comment but feel that we have addressed these points in the discussion section.

To summarize these points: In the second paragraph (line 384-393), we compare our results with other studies concerning psychological outcomes during the COVID-19 pandemic.; In the third paragraph (line 394-420), we compare our results with the results from other studies investigating the psychological response to hospitalization in several settings, including those during the MERS and SARS outbreaks.

7. Educational level is added to the regression models as a linear variable instead of dummy variables. This is a categorical variable with unequal distances between categories. The resulting coefficient is therefore meaningless.

**R:** We agree with the reviewer and have revised the following to improve this:

1) We added information about the rationale for the possible predictors in the methods section.
2) We used mixed models instead of linear regression models to identify predictors for psychological distress and HRQoL.
3) We added the results from the univariate analysis of all possible predictors, and the full model, in the supplementary information, including betas and p-values.

With regard to the education level variable: we used a dummy instead, and a beta of each category within the variable is presented in the tables.

8. The description of the measured used in the study is not sufficient. Please add range of scales and reliability statistics within your sample. Also elaborate on operationalization of other variables such as demographics. For instance, how is ethnicity operationalized? If more than two categories are used in the regression (such as native and non-native) see my previous comment as well.

**R:** We agree with the reviewer that the description of the questionnaires used in this study was limited. We therefore expanded the description of the questionnaires, including the ranges. The reliability of the validated questionnaires used, have been extensively reported previously and we refer to these papers in the method section.

With regard to the demographics, we extracted this information from the electronic patient records and used variable definitions as used for the ISARIC eCRF, as outlined in method section. For table 1, we have used the ethnicity as documented in the electronic patient record, and categorized ethnicity into ‘Caucasian’, ‘Negroid’, ‘Surinamese/Hindustan’, ‘Arab, not specified’, ‘Turkish’, ‘Moroccan’, ‘Other’, and ‘Unknown’. For analyses, we categorized these into ‘Caucasian’ and ‘non-Caucasian’.

9. No distinction is made in the regression analyses between the subgroups. Theoretically it would be interesting to distinguish the COVID and non-COVID patients. Ideally this would be done using a multilevel model to determine the impact of group membership on the influence of predictors, but the samples are probable too small for this.

**R:** We have revised our analysis and used mixed models. We have added ‘COVID-19’ and ‘ICU Admission’ as predictors, and only added these to the full model if the univariate analysis had a p-value below 0.10. As such, the predictive value, if present, can be extracted from the multivariate mixed models presented in the supporting information.

10. I think the statement on p16 that “Collectively, this suggest that participating patients are grossly representative for the total cohort” is too bold. Your response rate is quite low and examining patient records do not inform you how they would have scored on the outcome measures. A nice idea to ask a small sample of non-responders to respond anyway, but self-selection here is of course also a factor. You could consider comparing outcomes between the full responders (T1 and T2) and those that responded at T1 only.

**R:** We value the suggested outcomes comparison between full responders and those responded at T1 or T2 only as added analysis to address the non-responders’ issue. The results of that comparison is added as Table S8. In summary, the outcomes of full responders versus responders of one time-point only are similar, both for the one- and three-months’ time-point. Moreover, baseline demographic and treatment-related characteristics of these groups were also largely comparable (see Table S7).

We added the following:

“*In addition, both baseline demographics and treatment-related characteristics were similar for full responders and those who responded at one month or three months only (S7 Table). No major differences in psychological outcomes and HRQoL were observed between full responders and single time-point responders (S8 table).”* (Results, page 22, lines 373-376).

Notwithstanding, we agree with the reviewer that the sentence remains too bold, and we rewrote the sentence;

*“…, and in a sample of non-responders’ psychological distress was roughly similar to responders. Collectively, our cohort appears grossly comparable with the overall population at baseline and for psychological outcomes. Baseline characteristics (demographic and treatment-related factors) were similar for non-responders, responders, and a sample of non-responders willing to respond once. In addition, psychological outcomes and HRQoL were comparable for responders and the sample of non-responders willing to respond, and for responders at both time-points as compared to responders at 1 time-point only.”* (Discussion, page 25, lines 454-459).

Minor points:

1. I prefer a table where all primary and secondary outcomes can be seen, instead of a series of boxplots (which seem rather archaic). Having to look up a long list of supplementary tables is not helping your readers understand your work. especially as the tables put in the supplementary section are core study results. Readers are now forced to resort to the long summary of the results in the text, which do not give all information. I propose to put them into the article, and to merge them, and move the box-and-whisker plots to the supplementary section for the fans. This also means shortening the results section as not all results in the table then need to be described in text.

**R:** We fully agree with the reviewer’s point and have discarded the boxplots, and instead used tables to present our primary and secondary outcomes. We have focused the results section by shortening it, only described relevant outcomes, and referred to the tables for less relevant outcomes.

2. The Venn-diagrams look nice, but also add little. Please put these in the supplementary section as well.

**R:** We agree and discarded the Venn-diagrams, because in our new analysis and results section, we do not look into the co-presence of probable PTSD, anxiety, and depression.

3. There is no table for your regression analyses, not even in the supplements! Please add these (to the article, not the supplementary section).

**R:** We have added the tables of the mixed model analysis to identify predictors for both the severity of psychological distress (Table 4) and HRQoL (Table 5) to the manuscript, and added the tables with the results of the mixed models for the prediction of the prevalence of probable PTSD, anxiety, and depression to the supplements (S3. Table).

4. Please check for spelling and grammar errors such as “Predictors off psychological outcomes” on page 12. Or also see comment 10 these suggest/this suggests.

**R:** We apologize for this untidiness and copy-edited the entire manuscript.

5. When describing outcomes (especially when tables are not included) please add direction of association. E.g.: does having had a job before hospitalization lead to higher or lower psych symptoms?

**R:** We apologize for this untidiness and agree that this limits the comprehensibility or our results. We therefore added for each predictor whether it increased or reduced the outcome and added the direction by adding the betas.

6. Page 3: “the pandemic peak”. Is no longer applicable as there are 2 peaks at this point in time.

**R:** We agree and added ‘first’ to the sentence.

7. Provide a benchmark for HRQOL. You state it is low, but what are reference levels for for instance the general population?

**R:** We agree that this information was missing, and added the following to the results:

“*Based on the distribution of age in our cohort, the mean TTO score of the Dutch general population is 0.852 (37). A TTO score below 0.852 is considered poor, and a TTO score above 0.852 good.*” (Methods, page 8, lines 145-147)

“*Based on these scores, a mental and physical component score (MSC, PCS) can be computed for which the mean in the general population will be 50. A MCS or PCS score below 50 is considered low, and a MCS or PCS score above 50 is considered good (39).*” (Methods, page 8, lines 152-154)

**Response to Comments raised by Reviewer #3:**

Reviewer #3: The study topic “Psychological distress and health-related quality of life in patients after hospitalization during the COVID-19 pandemic: a single-centre, observational study” is relevant and interesting in this era of COVID-19 but is not clearly reported. I thank the authors for this good research, however many things need to be worked on to reach a level of publication

**Comments to the authors;**

**“Psychological distress and health-related quality of life in patients after hospitalization during the COVID-19 pandemic: a single-centre, observational study”**

The study topic is relevant and interesting but is not clearly reported.

**Introduction**

1. The introduction is too shallow. On top of what is written, the methods of psychological distress measurements used in this study need to be briefly described (i.e PTSD, depression, and anxiety measures using the Impact of Event Scale – Revised (IES-R) and the Hospital Anxiety and Depression Scale (HADS)). Briefly discuss as well how to measure health related quality of life using EQ-5D and RAND-36. This brief description should be done in a very concise and understandable manner.

**R:** Similar comments were raised by the academic editor and the other reviewers.

In short, we have expanded the introduction, and added more background information. We have described the questionnaire used in this study, including the cut-off values and ranges, more thoroughly in the methods section.

In the Introduction, we added the following:

“*Symptoms of the infectious disease caused by the SARS-CoV-2 virus, coronavirus disease-19 (COVID-19), vary from mild respiratory and gastro-intestinal symptoms to severe acute respiratory failure, requiring respiratory support in an Intensive Care Unit (ICU) (2-4).*“ (Introduction, page 4 lines 64-66)

*“; symptoms of depression and anxiety occur in 12-67% of hospitalized patients; symptoms of post-traumatic stress disorder (PTSD) in 19%-45% (8-15). Survivors of ICU treatment are known to be at risk for psychological impairments including symptoms of depression, anxiety and PTSD. These symptoms occur in up to 20%-67% of ICU survivors (16-21). Post-hospitalization psychological impairments negatively impact on the ability to return to work and the health-related quality of life (HRQoL) (13, 17, 18, 20). ”* (Introduction, page 4, lines 77-81)

*“The risk of psychological impairments is expected to be even higher in patients hospitalized during the COIVD-19 pandemic due to the exceptional circumstances and uncertainties (22, 23).“* (Introduction, page 4, lines 82-83)

In the Methods, we added the following:

“*The IES-R is a validated 22-item questionnaire that quantifies the subjective distress a person is experience after a traumatic event (32). The IES-R yields a total score, ranging from 0-88 (higher scores indicating more severe symptoms), and subscale scores can be calculated for symptoms of intrusion, avoidance and hyperarousal.*” (Methods, page 7, lines 132-135)

“*The HADS is commonly used to determine the levels of anxiety and depression that a person is experiencing (34). The HADS is 14-item scale that generated ordinal data. Seven of the items relate to anxiety and seven relate to depression. The HADS yields a depression and anxiety sum score, ranging from 0 to 21 with higher scores indicating more severe symptoms.*” (Methods, page 7, lines 135-138)

*“The EQ-5D measures the HRQoL on five dimensions (mobility, self-care, usual activities, pain/discomfort and anxiety/depression), by which the weight of a health state can be computed into an EQ-5D Time Trade Off (TTO) score. This score ranges from – 0.446 (worst quality of life) to 1.000 (best quality of life). Additionally, patients score their current subjective health state on a visual analogue scale (the EQ-VAS), ranging from 0 (worst health imaginable) to 100 (best health imaginable) (36, 37). Based on the distribution of age in our cohort, the mean TTO score of the Dutch general population is 0.852 (37). A TTO score below 0.852 is considered poor, and a TTO score above 0.852 good.”* (Methods, page 7, lines 141-147)

“*The RAND-36 is a 36-item, self-reported survey of HRQoL, and consists nine scales scores, which are the weighted sums of the questions in their section (38). Each scale is directly transformed to a scale ranging from 0 (worse score) to 100 (best score) on the assumption that each question carries an equal weight. The nine scores are; physical functioning, social functioning, physical role limitations, emotional role limitations, mental health, vitality, pain, general health, and health change. Based on these scores, a mental and physical component score (MSC, PCS) can be computed for which the mean in the general population will be 50. A MCS or PCS score below 50 is considered low, and a MCS or PCS score above 50 is considered good (39). Resumption to work and financial decline due to the hospitalization were was additionally assessed using self-composed questionnaires. Patients were asked for their psychiatric history and could choose whether or not to answer. Baseline characteristics were extracted from electronic patients’ records.*” (Methods, page 8, lines 148-157)

2. The problem statement of this study is not clearly stated: i.e “ Data from the previous SARS and MERS epidemics support this concern and more recent studies suggest that up to 50% of COVID-19 patients suffers from psychological distress up to one month after hospital discharge”. Looking at the results of this study (i.e of one of the results; “patients admitted for other illnesses more suffered psychologically than COVID-19” patients) , I was expecting to read a statement contradicting this to support your study results.

**R:** We agree that more elaboration is needed, and added the following sentence in the introduction section:

*“However, it is unknown whether this psychological response is attributed to COVID-19 or hospitalization during a pandemic, and whether the psychological response differs between specific subsets of patients, such as non-COVID-19 and ICU patients.*” (Introduction, page 4-5, lines 85-87)

We thereby question whether the high incidence of psychological distress during the previous SARS and MERS epidemics, and the incidence of psychological distress in COVID-19 patients is attributed to COVID-19, or to the pandemic circumstances.

3. The title, the aim of the study and results do not link well. Authors also say “Additionally, we tested the hypothesis that the psychological distress was higher in COVID-19 confirmed patients, and in those treated in ICUs”. Where is this hypothesis? Why the authors talk about an hypothesis which is not stated in the study?

**R:** We agree that a rationale for this hypothesis was lacking. With the revised introduction, we hope that it has become clearer why we conducted the study. We also described the aim of the study more clearly:

"*In this study, we therefore aimed to quantify the psychological distress, i.e., symptoms of PTSD, anxiety, and depression, up to three months post-discharge in patients hospitalized during the first pandemic peak with symptoms suggestive of COVID-19, to determine their HRQoL and work resumption, and to explore predictors for psychological distress and HRQoL. Additionally, we tested the hypothesis that examined whether psychological distress was more prevalent or more severe in COVID-19 confirmed patients, and in those treated in ICUs.*” (Introduction, page 5, lines 88-92)

**Materials and methods**

1. “**Study design and setting**”; when I read this heading, I expect to get information on study design and setting only. More information given here is about ethical consideration. For “study setting”, one should expect to read more of the description of the location, department, environment, infrastructure, etc… of the study place. The authors can either name this heading “**study design and ethical consideration**” or modify the content to suite the heading, and shift the content to an ethical heading. **NB: Please read more on study setting.**

**R:** We thank the reviewer for this useful comment. Accordingly, we have added additional information on the setting of the study:

“*This period coincided with the first COVID-19 peak. During this period, several protective measures were taken in the hospital, such as the prohibition of visiting hospitalized patients and strict isolation of suspected patients until COVID-19 was ruled out.*” (Methods, page 6, lines 104-106).

Additionally, we added an ‘Ethics approval”:

“**Ethics approval**

The study protocol was approved by the Institutional Review board of the Franciscus Gasthuis & Vlietland and deemed not to fall under the Medical Research Involving Human Subjects Act (WMO) (S1. Registered study protocol). The need for written informed consent was waived. Patients in our hospital are actively informed about the use of their anonymous data in research activities and can object against the use their data. No data were used of patients who objected against this use. The study was registered at TrialRegister.nl (registration number: [NL8882](https://www.trialregister.nl/trial/8882)). The reporting of this study follows the Strengthening the Reporting of Observational Studies in Epidemiology (STROBE) guideline (S2. STROBE Checklist) (31).*“* (Methods, page 6, lines 94-101)

**2.“participants”;** in this section, the inclusion and exclusion criteria are not clear. “the presence of respiratory symptoms (e.g., dyspnoea, coughing, sore throat, rhinorrhoea, saturation 24/minute) and/or gastro-intestinal symptoms (e.g., diarrhoea or vomiting) with a duration ≥24 hours”. The study inclusion and exclusion should be clear. The authors should not give examples of symptoms but should clearly state which symptoms constituted inclusion criteria and which constitute exclusion criteria. I suggest a heading “**Inclusion and exclusion criteria**” **or Eligibility criteria**.

**R:** We agree with the reviewer, and more clearly stated the inclusion- and exclusion criteria in the manuscript. It now reads as follows:

“*Eligible patients were aged ≥18 years and hospitalized between March 16 and April 28, 2020, with symptoms suggestive of COVID-19, defined as the presence of respiratory symptoms (dyspnea, coughing, sore throat, rhinorrhoea, saturation <94% or respiratory rate >24/minute) and/or gastro-intestinal symptoms (diarrhea or vomiting) with a duration ≥24 hours, who survived until one month after hospital discharge. Patients who were unable to understand the Dutch language or did neither have a formal home address nor e-mail address were excluded.”* (Methods, page 6, lines 111-116)

**3. “Procedures”;** With this heading two important considerations come up: (1) Data collection tools/instruments; after reading this study, 4 questionnaires and one data collection tool are supposed to be used yet none is described in this section. Please discuss briefly these tools;” (Impact 93 of Event Scale – Revised (IES-R), the Hospital Anxiety and Depression Scale (HADS), EQ-5D, RAND 36 and demographic data collection tool”

**R:** In accordance with the academic editor’s and the previous reviewers’ comments, we have added a more detailed description of the questionnaires and how we obtained baseline demographics and treatment-related characteristics.

Authors say “Patients who were unable to understand the Dutch language or did neither have a formal home address nor e-mail address were excluded”. The dutch language is another issue on data collection tool used to collect data. It should come clearly what was the language of the questionnaires. “did the authors used a standard questionnaire in Dutch”? If yes, clarify how this was translated. If the original questionnaire was in English, clarify how it was translated in Dutch then back in English for data entry.

**R:** We agree with the reviewer that this should be stated within the manuscript, and therefore added:

“*We used validated Dutch translations of the IES-R, HADS, RAND-36, and EQ-5D.*” (Methods, page 8, lines 166-167)

(2) **Sampling procedure**; it is not clear how the sampling procedure was done. Random sampling? Convenient sampling?.....The author needs to explain how the sampling procedure was done and clarify the sample size as well.

**R**: As stated in the “Participants” and “Procedures” sections of the Methods section, we invited all patients admitted with symptoms suggestive of COVID-19 to participate in the study and included patients who responded to these questionnaires. As such, convenient sampling was used. We added the following sentence into the method section:

“*Convenient sampling was used. All patients admitted between March 16 and April 28, 2020, and who responded to one of the two follow-up assessments were included*” (Methods, page 7, lines 125-126)

(3) It is also not clear who collected the data. Were authors collected data themselves? or trained research assistants to collect data.

**R**: All data was collected by two researched from the study team (Johan H. Vlake and Sanne Wesselius). We added the following to the methods:

“*All data was collected by members of the study team (JV and SW)*.” (Methods, page 8, line 167)

**4.”Outcomes”;** In this section, it is expected to explain how authors have reached the mentioned outcomes. I.e for EQ-5D questionnaire, the author must clarify which **published algorithm** used to allow **a CROSSWALK** to measure utility. “ The questionnaire should score using a **published algorithm** **based on community survey in the country or neighboring country using standard Gamble, Time trade off or visual analogue scale(VAS)”.**

**R:** We agree with the reviewer that the explanation given in the methods section regarding the outcomes measures was limited, and therefore we elaborated the description of how the sum scores, VAS score, TTO score, and summary scores were computed. In addition, we referred to the appropriate literature supporting these methods.

**5.”Statistical analysis”;** The analysis of this study is well done but poorly reported. i.e firstly, the author reported “ correlations between psychological outcomes and baseline/treatment characteristics were analyzed using a Spearman’s rho”. The authors should report the correlation coefficients. Secondly, the authors said “Possible predictors for psychological outcomes were analyzed using a **multivariate linear regression model.** Correlations with a p-value below 0.10 were included in a **multivariate linear regression model** to identify the predictor”. Please review this statement if this is what you wanted to say and rectify.

**R:** We agree with the reviewer that the statistical analysis was not properly described. We revised our analysis plan and rewrote this section accordingly. We used mixed models instead of general regression models and reported al betas and p-values for the univariate and multivariate mixed models in the supplementary information.

Concordantly, we added the following:

“*Differences in outcome measures between stratifications were analyzed using linear or logistic regression models for continuous and categorical outcomes, respectively, adjusting for baseline differences between groups, in which the ‘stratification’ and at baseline differing characteristics served as independent variables.”* (Methods, page 9, lines 172-174).

“*To identify possible predictors for psychological symptomatology or quality of life, we first conducted a univariate linear or logistic mixed model with possible predictors as independent variables (S1. Table). Variables with a p-value ≤0.10 were added in the multivariate linear or logistic mixed model to determine predictors.*” (Methods, page 9, lines 180-182)

**Results**

1.In the participants section, the results are not clearly explained. It is hard to understand this paragraph; “A total 123 of 282 individuals participated (45%; Fig 1); 252 at one month (40%) and 201 of 365 (55%) at three months post discharge. Non-participating patients either declined participation (n=262) or did not respond (n=79)”. It is also difficult to understand the response rate of the 4 questionnaires distributed twice (at 1 month and 3 months). Please restructure this section by shading more light to this section.

**R:** We restructured this section to make this more comprehensible:

“*From March 16 to April 28, 2020, 623 patients were admitted with symptoms suggestive of COVID-19, of whom 282 patients participated (45%); 252 at one month and 201 at three months post-discharge (Fig 1). The last patient was discharged June 14, 2021, and follow-up lasted until three months after (September 14, 2020). Non-participating patient either declined participation (n=262) or did not respond to the questionnaires or to the reminding phone calls (n=79).“* (Results, page 10, lines 194-198)

In addition, we changed figure 1 to make it more comprehensible.

2. It is also not clear when the data collection was done. Please clarify these i.e; in the Study design section; the authors say “This single-centre, observational cohort study was conducted in the Franciscus Gasthuis & Vlietland hospital in Rotterdam, the Netherlands, from March 16 to September 14, 2020”. And in Participants section; the authors say “Eligible patients were aged ≥18 years and hospitalized between March 16 and April 28, 2020”. And in results section, the authors say as well “From March 16 to April 28, 2020, 623 eligible patients were admitted with symptoms suggestive of COVID-19”.

**R:** We understand the confusion with how we described this. Patients were included if they were admitted to the hospital between March 16, 2020, and April 28, 2020. Since the follow-up ran to three months after hospital discharge, the entire study period lasted from March 16, 2020, till three months after the hospital **discharge** of the last included patients, which was on June 14, 2020, and thus follow-up of the last participant ran until September 14, 2020.

To make this more understandable, we added the following sentences:

“*The last patient was discharged June 14, 2020, and follow-up lasted until three months after (September 14, 2020).*” (Methods, page 7, lines 124-125)

“*The last patient was discharged June 14, 2021, and follow-up lasted until three months after (September 14, 2020).*” (Results, page 10, lines 195-196)

**3. My Suggestion on how the results should be presented in results section**

a. Introduce the results section and present demographic characteristics in a table, comment your results from the table

**R**: We have restructured the results, and start with the inclusion and responses of participants, followed by a section regarding demographics, in which we primarily comment on the results of Table 1, which comprise the baseline demographics and treatment-related characteristics of our cohort.

b. Present psychological distress results in a table or a clear figure, then comment the table or figure in an understandable manner. Show and comment on any P-value

**R:** We discarded the boxplots, and present our psychological outcomes in tables only. In the results section, we focused on the most relevant data, discarded the less relevant and refer to tables for more extensive data insight.

c. Present the health related quality of life in a table or a figure, comment the results. Show and comments P-values if any

**R:** We have discarded the boxplots of the HRQoL and replaced them by tables. In the revised text, we focused on commenting on results which we found most relevant and referred to the tables for other study results.

d. Present the predictors of psychological outcomes and quality of life in a table showing the regression results and P-values, correlation coefficients and comment the table.

**R:** We have added tables regarding the predictors of the severity of psychological outcomes and predictors or HRQoL in table 4 and table 5, including betas and P-values, and only described variables that significantly predicted outcomes. The predictors for the prevalence of probable PTSD, anxiety, and depression are depicted in Table S3 in the supplementary information.

**Discussion and references;** I cannot comment on discussion and references for now, if results are not presented in an understandable manner

**R:** We understand that the reviewer could not comment on the discussion at this stage. We have revised the discussion based on the revisions in the introduction, methods, and result section, and invite the reviewer to comment on the discussion section.
