# Supplementary material for: Psychological distress and health-related quality of life in patients after hospitalization during the COVID-19 pandemic: A single-center, observational study
Source: PLoS One. 2021 Aug 11;16(8):e0255774. doi: 10.1371/journal.pone.0255774 (PMC8357130; doi:10.1371/journal.pone.0255774)
Supplement: Attachment — Submitted filename: PONE-D-20-38679R1.docx [file pone.0255774.s016.docx]

**Second review: Psychological distress and health-related quality of life in patients after hospitalization during the COVID-19 pandemic: a single-center, observational study**

I want to thank the authors for the good job done, especially on methodology. There is a significant improvement on the quality of the manuscript. However, some work need to be done to allow the paper to be published

**Introduction**

1. This section was reviewed partially. “This paper’s aim is to quantify psychological distress and to determine their HRQoL”. The introduction needs a section to briefly discuss few different methods to assess mental health and HRQoL. This section will help non expert readers to understand and also give a good flavor to the reader

**Methodology**

Authors have made significant progress in term of reporting methodology. However, data analysis part is not well reported;

it is not clear in the analysis which method used at each level, for example;

2. Line 172; “Differences in outcome measures between stratifications were analyzed using linear or logistic regression”. Clarify where you used linear and where you used logistic regression and clarify the logistic model used (i.e function or Equation). For logistic regression (binary outcomes), I was expecting to read the results with Odds ratios and confidence intervals. Please explain this!

3. Line 185-186 “Psychological outcomes of randomly selected non-responders were compared with those of responders using a linear or logistic regression model”. It is not clear where applied linear regression, where applied multiple and where applied logistic regression. Please clarify these by showing which statistical model used (i.e function or equation).

4. Please discuss with your analyst if there is a possibility of using “ Time to event outcomes regression” (cox proportional hazards model) and calculate hazard ratios for ICU versus non-ICU and COVID-19 versus non COVID-19 in the development or reduction of psychological distress from the time of discharge to one month time follow up!

**Health related quality of life;**

5. Clarify how you calculated utility scores and health states, then how did you do the crosswalk for the health states?

6. Explain how you calculated the mean utility scores

7. Explain how you estimated QALYS lost due to psychological distress

**Results**

All relevant or significant results (i. e significant P-values) in the tables in this section must be briefly commented below each table.

8. For example, “Table1: Baseline demographics and treatment-related characteristics of study population”. Where are comments of this table?

9. Table2 “Psychological outcomes throughout follow-up” is confusing because it is not commented. I don’t see the relevance of this table. And if am not wrong, no significant results are reported in this table. And then, the bottom of this table reads logistic regression. With logistic regression results, one would expect to be reported Odds ratios (ORs) and confidence intervals. I suggest you remove this table because the results are confusing and are not explained therefore it does not add value unless if it is clearly commented.

10. Table3, 4 and 5 are relevant but are not commented. Please comment important or significant results in these tables

**Discussion**

I am still not able to comment on discussion; results need to be worked on before reviewing the discussion part.

11. Intext citations and references don’t follow PLOS ONE guidelines. Please check on PLOS ONE referencing guidelines
