# Supplementary material for: Psychological distress and health-related quality of life in patients after hospitalization during the COVID-19 pandemic: A single-center, observational study
Source: PLoS One. 2021 Aug 11;16(8):e0255774. doi: 10.1371/journal.pone.0255774 (PMC8357130; doi:10.1371/journal.pone.0255774)
Supplement: Attachment — Submitted filename: Response to the reviewers.docx [file pone.0255774.s017.docx]

**Response to the handling editor:**

Dear Dr. Vlake,

Thank you for submitting your manuscript to PLOS ONE. After careful consideration, we feel that it has merit but does not fully meet PLOS ONE’s publication criteria as it currently stands. Therefore, we invite you to submit a revised version of the manuscript that addresses the points raised during the review process.

I have read your revised manuscript with pleasure and noticed that your manuscript improved a lot. You are one important step further. There are several things that must be addressed now before I can make a final decision. You therefore have to address the helpful comments of reviewer 3 (see below) and my specific comments below. As you will see, must comments concern clarifications, corrections and suggestion to omit certain sections. The requested revision is somewhere between a minor and major revision (PLOS ONE does not have that option).

Dear Peter van der Velden,

We really appreciate that you are currently handling our manuscript and that you have given us the opportunity to thoroughly revise our manuscript.

The suggestions, concerns, and comments raised by you and reviewer 3 did further improve our manuscript and we have made point-by-point responses to your and the reviewer’s comments below.

For the referral to certain pages or lines within the manuscript; all referrals are based on the submitted marked-up manuscript, in which the changes made during the revision are highlighted.

My comments are:

1.	Please update your literature, in intro as well as in discussion, with the very recent study by Taquet et al., 2021 ([https://doi.org/10.1016/S2215-0366(21)00084-5)](about:blank)

**R:** We agree with the editor that an update of published articles is in place for both the introduction and discussion sections. We therefore updated the introduction and discussion, as suggested by the editor.

We added the following text into the introduction and discussion section:

“*The uncertainties and measures surrounding the pandemic raised concerns of increased psychological distress in non-hospitalized citizens (20). Since measures taken in hospital were more drastic, concerns of the mental well-being of hospitalized patients were even higher (21, 22). Data from the previous SARS and MERS epidemics support this concern and early studies suggest that up to 50% of COVID-19 patients suffer from psychological distress up to two months after hospital discharge (23-28). More recently, Taquet et al. estimated that the risk for psychiatric sequelae is higher in COVID-19 patients and in those admitted to ICU using electronic health records data. Complementary observational studies are needed to add direction as to whether these risks may be attributed to COVID-19 or hospitalization during a pandemic, and corroborate on the different risks between specific subsets of patients, such as non-COVID-19 and ICU patients (29).*” (Introduction, page 3, lines 63-71)

*“In a recent large retrospective study, COVID-19 was indeed associated with a higher risk of psychiatric outcomes as compared to influenza and respiratory tract infections (29). In our cohort however, psychological distress was largely similar between non-COVID-19 patients with respiratory symptoms and COVID-19 patients, and we did not observe COVID-19 positivity to be a predictor for severity of PTSD, anxiety, or depression. As such, our data collectively suggest that COVID-19 on its own has no major influence on psychological outcome. The contradictory findings of the Taquet and our study will largely be explained by the difference in study design (retrospective cohort vs. prospective observational cohort), the different outcome measures used (ICD-based diagnoses vs. questionnaire-based outcome) and comparing the covid-19 cohort with other control populations (influenza and respiratory tract infection vs. patient with symptoms suggestive of COVID-19 but COVID-19 PCR negatives).*“ (Discussion, page 25, lines 369-378)

2.	You examined several predictors of mental health problems and quality of life. In the introduction section explain much more in detail which predictors you want to examine and why you specifically want to examine these predictors. You can use the content of Table S1 for this purpose and omit this tables from the supp. materials. Explain too for which variables you control for when comparing groups in mental health problems and quality of life (medical conditions etc.).

**R:** We agree with the editor, and added the following to the introduction concerning the predictors examined:

“*Illnesses requiring hospitalization, particularly those requiring intensive care unit (ICU) treatment, are known to negatively impact post-discharge psychological well-being; symptoms of depression and anxiety occur in up to 67% of hospitalized patients and symptoms of post-traumatic stress disorder (PTSD) in up to 45% (2-15). This post-hospitalization psychological distress negatively impacts the health-related quality of life (HRQoL) (7, 11, 12, 14). Both demographic characteristics, such as female gender, lower educational level, unemployment and non-western ethnicity, and treatment-related characteristics, such as duration of admission and severity of the disease, are known to be negatively associated with psychological well-being of patients (2, 8, 16-19).*

*The uncertainties and measures surrounding the pandemic raised concerns of increased psychological distress in non-hospitalized citizens (20). Since measures taken in hospital were more drastic, concerns of the mental well-being of hospitalized patients were even higher (21, 22). Data from the previous SARS and MERS epidemics support this concern and early studies suggest that up to 50% of COVID-19 patients suffer from psychological distress up to two months after hospital discharge (23-28). More recently, Taquet et al. estimated that the risk for psychiatric sequelae is higher in COVID-19 patients and in those admitted to ICU using electronic health records data. Complementary observational studies are needed to add direction as to whether these risks may be attributed to COVID-19 or hospitalization during a pandemic, and corroborate on the different risks between specific subsets of patients, such as non-COVID-19 and ICU patients (29).*” (Introduction, page 3, lines 56-71)

We however believe that Table S1 has added value for the reader who is interested in an overview of the literature supporting our choice of predictors, and we therefore would like to suggest to keep Table S1 as part of the Supplements.

With regard to the controlling variables in the comparison between COVID-19 and non-COVID-19 patients, and the comparison between COVID-19 ICU and COVID-19 non-ICU patients; firstly, we better described this in the Statistical analysis section of the methods:

“*Differences in outcome measures between stratifications were analyzed using simple linear or logistic regression models, to adjust for at baseline differing characteristics, for continuous and categorical outcomes, respectively. We therefore first performed a simple univariate linear (for continuous outcomes) or logistic (for categorical outcomes) regression analyses, in which all at baseline differing characteristics were analyzed one-by-one. Variables that were associated with the outcome, i.e., a p-value ≤0.10, were added as independent variables to the simple multivariate regression models and were as such adjusted for.*“ (Methods, Statistical analysis, page 9, lines 195-200)

Additionally, we described for which variables we adjusted in the legends of the Tables.

3.	You examined patients from one hospital. Did you control for the possibility that patients can be identified based on the content of the Tables? If yes, add if and how the anonymity was ensured.

**R:** We understand the concerns of the editor. We have re-checked whether the content of our tables could lead to the identification of individual patients in our study and omitted categorical variables from the supplementary dataset that were only present in one patients of a stratifications and could as such lead to the identification of an individual patient. These included ethnicity, psychiatric history outcomes, treatment code before discharge, and presence of several comorbidities.

For other tables; we only present the median values or regression outcomes of the entire population and stratifications. In none of the stratifications, nor in the entire cohort, probable PTSD, anxiety, or depression was reported by only 1 patient. As such, patients cannot be identified based on these tables.

4.	Add Cronbach’s alphas for all scales for COVID and non-COVID patients (IES-R, etc.)

**R:** We agree, and added the following for the Statistical analysis section of the methods:

“*The internal reliability of all questionnaires used (IES-R, HADS, EQ-5D and RAND-36) were analyzed using Cronbach’s alpha and all showed a high internal reliability (Table S2).*” (Methods, Statistical analysis, page 9, line 190-191)

Additionally, we added the following table to the supplements:

| **S2 Table. Reliability of the impact of event scale-revised, the hospital anxiety and depression scale, the short-form 36, and the EQ-5D in the entire population and stratifications.** | | | | | |
| --- | --- | --- | --- | --- | --- |
|  |  | **Total cohort** | | **COVID-19 cohort** | |
| **Questionnaires** | Total cohort | COVID-19 | non-COVID-19 | ICU | non-ICU |
| Impact of Event Scale – Revised (IES-R) | 0.937 | 0.939 | 0.922 | 0.912 | 0.945 |
| Hospital Anxiety and Depression Scale (HADS) | 0.935 | 0.934 | 0.933 | 0.923 | 0.937 |
| Short-Form 36 (SF-36) | 0.949 | 0.949 | 0.948 | 0.947 | 0.950 |
| European Quality of Life – 5 dimensions (EQ-5D-5L) | 0.834 | 0.822 | 0.837 | 0.825 | 0.821 |
| Values represent the Cronbach’s Alphas, and give an indication of the internal reliability of the questionnaire.  A Cronbach’s alpha ≥ 0.9 is considered excellent, a Cronbach’s alpha ≥ 0.8 is considered good. | | | | | |

5.	Please replace the subheading Outcomes with Measures. Table 1 contains many variables that were not described in the measures section. Add them in the measures section. It may help the reader if you add sub-subheadings to the measures section (Psychological distress, Quality of life, Cause of admission, etc). In the measures section all study variables must be mentioned/explained/clarified. For instance table 2 shows that you assesses several categories psychiatric history (psychologist, etc), but this was not clarified in the text. Check all variables.

**R:** We apologize for the untidiness. We have now described all outcomes that were collected during the study, replaced the subheading Outcomes with Measures, and added subheadings to the section.

The section now reads:

“***Measures***

*All data were gathered using the International Severe Acute Respiratory and emergency Infection Consortium (ISARIC, Oxford, United Kingdom) and Franciscus Corona Registry in Castor Electronic Data Capture (Castor EDC, Amsterdam, the Netherlands). The ISARIC database in an international initiative to collect baseline demographics and treatment-related characteristics of all patients admitted to the hospital with respiratory symptoms during the SARS-CoV-2 pandemic. The Franciscus Corona Registry is a local addition to the ISARIC database, in which variables that were not collected in the ISARIC database, but were required for the current trial, were collected. All data was collected by members of the study team (JV and SW).*

***Baseline demographics and treatment-related characteristics***

*The following baseline demographics were retrieved from electronic healthcare records: age (years), ethnicity (Caucasian, black, Surinamese/Hindustan, Arab (not specified), Turkish, Moroccan, others, or unknown), sex at birth (male or female), body mass index (kg / m2), comorbidities (yes/no: hypertension, chronic cardiac disease, chronic pulmonary disease, asthma, tuberculosis, chronic kidney disease, mild liver disease, moderate liver disease, chronic neurological disease, dementia, chronic hematologic disease, diabetes type I or II, rheumatologic disorder, malignant neoplasm; total number of comorbidities), smoking status (yes, never smoked, former smoker, unknown). Patients were asked about their educational level (i.e., elementary school, high school, intermediate vocational education, higher professional education, university education) and employment characteristics (employed before hospitalization yes/no, weekly work hours before admission, weekly work hours after discharge, healthcare worker yes/no). Additionally, we asked patients about their mental history, i.e., whether they had encountered psychological impairments in the past 5 years and whether they were treated for these impairments by a psychologist, psychiatrist or had received medication. Patients were free to decide whether or not to answer the questions regarding their mental history.*

*The following treatment-related characteristics were retrieved from electronic healthcare records: cause of admission, treatment restrictions at the day of hospital admission, last registered treatment restriction before discharge, hospital length of stay, ICU admission (yes/no, length of stay), SOFA score at admission, P/F ratio at admission, S/F ratio at admission, oxygen therapy (yes/no, duration), non-invasive ventilation (yes/no, duration), invasive ventilation (yes/no, duration), prone positioning (yes/no, duration), tracheostomy (yes/no) and survival during follow-up.*

***Primary outcome: Psychological distress***

*The primary outcome was the prevalence and severity of probable PTSD, depression, and anxiety, assessed using validated Dutch translations of the Impact of Event Scale – Revised (IES-R) and the Hospital Anxiety and Depression Scale (HADS) at one and three months post-discharge.*

*The IES-R is a 22-item questionnaire that quantifies the subjective distress a person is experience after a traumatic event (31). The IES-R yields a sum score, ranging from 0-88 (higher scores indicating more severe symptoms), and subscale scores can be calculated for symptoms of intrusion, avoidance, and hyperarousal. The IES-R sum score was considered as the severity of PTSD, and an IES-R sum score above 24 was defined as probable PTSD (32).*

*The HADS is commonly used to determine the levels of anxiety and depression that a person is experiencing (33). The HADS is a 14-item scale that generates ordinal data. Seven of the items relate to anxiety and seven relate to depression. The HADS yields a depression and anxiety sum score, ranging from 0 to 21, with higher scores indicating more severe symptoms. The HADS anxiety and depression score will be considered as the severity of anxiety- and depression-related symptoms, respectively. A HADS depression or anxiety score above 8 was defined as probable depression or probable anxiety, respectively (33, 34).*

***Secondary outcome: Health-related quality of life***

*The secondary outcome was HRQoL. HRQoL was assessed using validated Dutch translations of the EuroQoL 5-dimensions-5-levels (EQ-5D) and the RAND-36 questionnaires at one and three months post-discharge.*

*The EQ-5D measures the HRQoL on five dimensions (mobility, self-care, usual activities, pain/discomfort, and anxiety/depression), by which the weight of a health state can be computed into an EQ-5D Time Trade Off (TTO) score. This score ranges from – 0.446 (worst quality of life) to 1.000 (best quality of life) and will be considered as the overall HRQoL. Additionally, patients score their current subjective perceived health state on a visual analogue scale (EQ-5D VAS), ranging from 0 (worst health imaginable) to 100 (best health imaginable) (35, 36). Based on the distribution of age in our cohort, the mean TTO score of the Dutch general population is 0.852 (36). A TTO score below 0.852 is considered poor, and a TTO score above 0.852 good.*

*The RAND-36 is a 36-item, self-reported survey of HRQoL, and consists nine scales scores, which are the weighted sums of the questions in their section (37). Each scale is directly transformed to a scale ranging from 0 (worse score) to 100 (best score) on the assumption that each question carries an equal weight. The nine scores are: physical functioning, social functioning, physical role limitations, emotional role limitations, mental health, vitality, pain, general health, and health change. Based on these scores, a mental and physical component score (MSC-36, PCS-36) can be computed for which the mean in the general population will be 50 with a standard deviation (SD) of 10. The MCS-36 score will be considered as the mental HRQoL and the PCS-36 will be considered as the physical HRQoL. A MCS-36 or PCS-36 score below 50 is considered low, and a MCS or PCS score above 50 is considered good (38).*

***Exploratory outcomes***

*We additionally explored predictors for the severity and prevalence of psychological distress and the HRQoL. These predictors were chosen based on previous literature, and included: age, gender, ethnicity, educational level, duration of admission, ICU admission, COVID-19 diagnosis and work before admission, and severity of disease in terms of SOFA score at the first day of COVID-19 suspicion (day of enrolment) (2, 8, 16-19). Literature supporting these predictors is depicted in Table S1 in the Supporting Information.”* (Methods, Measures, page 6-8, lines 109-176)

6.	Are there no cut-offs available for the HRQoL scales?

**R:** The editor is right that there are no cut-offs available for the HRQoL scales. As the EQ-5D and RAND-36 are used to quantify HRQoL, rather than measure the presence of a disease, there are no pre-specified cut-off values available. Therefore, we used the mean values of the MCS-36, PCS-36, and RAND-36 subscales of the general Dutch population, retrieved from validation studies, as reference.

7.	Line 155. Table 1 suggest that the ICU-nonICU comparisons were conducted among the COVID patients only (39+105=144). If this is correct, please clarify this better in this line (see also below).

**R:** The editor is right that we compared outcomes of COVID ICU and COVID-19 ICU survivors. To explain: only 2 patients were admitted to the ICU with another cause than COVID-19. As such, we decided to analyze differences between ICU and non-ICU patients only within the COVID-19 cohort.

To clarify this matter, we adjusted the first rows of Table 1:

| **Table 1.** Baseline demographics and treatment-related characteristics of study population. | | | | | | |
| --- | --- | --- | --- | --- | --- | --- |
|  | **Overall cohort** | | | **COVID-19 cohort** | | |
| **Overall**  **(n=282)** | **COVID-19** | **Non-COVID-19** |  | **ICU** | **Non-ICU** |  |
|  | **(n=144)** | **(n=138)** | **p-value** | **(n=39)** | **(n=105)** | **p-value** |

8.	Line 161. Please explain better the dependent variables in your analyses because there are several ways to examine potential predictors. I would like to suggest that you perform a multiple regression analyses with your distress and QOL variables at T2 as dependent variables (instead of the current analyses), and your proposed potential predictors as predictors including the corresponding distress/QOL variable at baseline. In this way you have also solved the problem that some variables were assessed at T2 (containing info over the period between T1 and T2). In addition, it offers the reader more info over the extent your variables predict distress/QOL.

**R:** We thank the editor for this comment, and in hindsight understand that we did not explain thoroughly enough how we conducted our analysis.

First, we want to stress that the T1 measurement was not a baseline measurement, but the first follow-up assessment at one month post-hospital discharge. As such, using the psychological distress and QoL outcomes at T1 as independent variables in our regression models will firstly disable us to draw any conclusions about the course of psychological distress, i.e., whether psychological distress improved or worsened during follow-up, and secondly will possibly disable us to find relevant risk factors, as these are expected to be correlated to the outcomes at T1 and therefore are likely to lose significance in the model.

Also, with regard to the concerns about the missing data; in our analysis, we used mixed effects linear regression models. These account for both inter-group differences, such as the stratifications and at baseline differing variables between stratifications, and intra-group differences, such as time after discharge. Using mixed effects linear regression models, slopes are computed for each individual patient, and the coefficient computed takes into account all of these slopes, and all data provided. As such, the model uses all data available instead of only participants who filled out both follow-up assessments, and thereby adjusts for missing values. Hence, we believe that the current analysis is more robust than the suggested analysis of the editor.

To clarify, we added the following to the Statistical analysis section of the Methods:

“*To identify possible predictors for the severity and prevalence of psychological distress and the overall, mental, and physical HRQoL, we first conducted univariate mixed effects regression analysis: a linear model for continuous outcomes and a logistic model for categorical outcomes. We used mixed effects regression models to adjust for intergroup (i.e., time) and intragroup differences (i.e., cohort and variables of interest). In these, the possible predictive variable served as independent variable one-by-one and the outcome of interest at both follow-up time points served as dependent variables. Secondly, all variables which showed a p-value ≤0.10 in the univariate mixed effects regression model were added to the multivariate mixed effects regression model to determine which variables significantly predicted the outcome. We report the coefficient [95% CI], which implies the estimated mean difference, for linear models, and odds ratios (ORs), including its 95% CI, for logistic models.*” (Methods, Statistical Analysis, page 9-10, lines 201-209)

9.	Line 162. What do you mean with logistic mixed model?: mixed effects logistic regression analyses?. What do you mean with “univariate linear”, correlations? (where multivariate linear = multiple regression analyses?)”. Please explain better what type of analyses you used for differences between groups and differences over time, and for predictors.

**R:** We apologize for the unclarity, and partially answered these questions in your previous comment. In the univariate mixed effect linear/logistic regression model, we assessed whether any association was observed between the possible predictor and the outcome of interest. Thereafter, all variables that showed a high correlation, i.e., a *p*-value ≤0.10, were added to the multivariate mixed effects regression model. Variables that showed a significant contribution to the multivariate mixed effects regression model were considered as independent predictors.

10.	Line 166. It is unclear what you meant with “Psychological outcomes of randomly selected non-responders were compared with those of responders using a linear or logistic regression model, adjusting for baseline differences between groups, in which the ‘stratification’ and at baseline differing characteristics served as independent variables”. Please clarify this better and especially “randomly selected non-responders”. You have two type of non-response: those not participating at all (N=341) and those participating at baseline but not at follow-up. Please clarify this better in the text with results of the analyses. Move the non-response section directly above the first results section.

**R:** We thank the editor for this comment, as we realize we did not explain clearly enough how we assessed the non-responders bias.

To clarify, to analyze non-responders bias, we asked non-responders, i.e., patients who had expressed a wish not to take part of this study or who had not returned any of the questionnaire, to fill out a single set of questionnaires approximately 1 month after the last questionnaire was sent. A total of 12 non-responders agreed, and the outcomes of these 12 non-responders were compared with the outcomes of ‘true’ responders to determine whether there were (substantial) differences in outcomes.

We agree that we did not formulate with clear enough, and changed this accordingly:

“*We randomly approached non-responders (who did not respond at both time-points) four months after hospital discharge, i.e., one month after sending the last questionnaire, and asked them to fill out a single set of questionnaires to analyze non-responder’s bias.*” (Methods, Procedures, page 6, lines 106-108)

“*Baseline characteristics of all non-responders were compared with those of responders using a Mann-Whitney U test for continuous variables and using a Fisher’s exact test for categorical variables. Psychological outcomes of randomly selected non-responders (who did not respond at both time-points) were compared with those of responders, and psychological outcomes of full responders were compared with those of partial responders (i.e., patients who only responded at one or three months), using simple linear or logistic regression models for continuous and categorical variables, respectively. In these, we adjusted for variables that were expected to confound the outcome and differed at baseline between stratifications as described above.*” (Statistical analysis, methods, page 10, lines 210-216)

Additionally, we moved the ‘Non-responder’s analysis’ section to the beginning of the result section.

11.	Line 169. “All data were gathered using the International Severe Acute Respiratory and emergency Infection Consortium (ISARIC, Oxford, United Kingdom) and Franciscus Corona Registry in Castor Electronic Data Capture (Castor EDC, Amsterdam, the Netherlands).” I do not see the relationship with statistical analyses and don’t understand what you want to say here (should it be moved to the measures section in which you describe which variables were extracted from both?).

**R:** We agree and changed accordingly, and moved this to the measures section:

“*All data were gathered using the International Severe Acute Respiratory and emergency Infection Consortium (ISARIC, Oxford, United Kingdom) and Franciscus Corona Registry in Castor Electronic Data Capture (Castor EDC, Amsterdam, the Netherlands). The ISARIC database in an international initiative to collect baseline demographics and treatment-related characteristics of all patients admitted to the hospital with respiratory symptoms during the SARS-CoV-2 pandemic. The Franciscus Corona Registry is a local addition to the ISARIC database, in which variables that were not collected in the ISARIC database, but were required for the current trial, were collected.*” (Methods, Measures, page 6, lines 110-115)

12.	Line 187. The scores and percentages in Table 1 seem an selection of Table S2. It was not clear to me how this selection was motivated. I expect that you help the reader by explaining this selection (something like “…..In table 1 the main characteristic of the study samples is presented and for a full overview of the characteristic we refer to S2 table”.

**R:** The editor is right that Table 1 is a selection of Table S2, and changed accordingly:

“*Table 1 depicts the most relevant baseline demographics and treatment-related characteristics; a full overview of all collected demographics and characteristics can be found in Table S8.*“ (Results, Participants, page 11, line 238-239)

13.	Line 190. Median scores were not introduced in the stat. analyses section. The relevance of the median scores in Table 2 and 3 was totally unclear to me. What is the relevance? I would like to suggest that you conduct mixed models / ANCOVA to examine differences in sum scores of the used scales (with M and SD in tables).

**R:** We apologize for this untidiness, and now described in the Statistical analysis section how variables will be presented:

“*Continuous variables are presented as mean, including its standard deviation (SD), if normally distributed, and as median, including its 95% range, if not normally distributed. Categorical variables are presented as absolute and relative frequency.*” (Methods, Statistical analysis, page 9, lines 178-180)

With regard to Table 2 and 3; we regret that the relevance of these tables was unclear, and a similar comment was raised by reviewer 3. Table 2 and 3 depicts our primary and secondary outcome measures; Table 2 represents the severity and prevalence of psychological distress, as measured at both follow-up moments. The P-values provided regarding the comparison between stratifications are calculated using simple linear and logistic regression models, which is largely comparable with an ANCOVA, to adjust for at baseline differing variables. The P-values regarding the change over time were calculated using mixed effects linear and logistic regression models. To clarify, we added the coefficients of the regression models to the Tables.

As the outcome variables presented in these tables were not normally distributed, we report medians rather than means. We can however still use regression models as long as the residuals of the model are normally distributed, and no other assumptions are violated.

To clarify this, we revised the legend of Table 2:

“*Descriptive statistics of the psychological distress outcomes, stratified by COVID-19 diagnosis and ICU admission. Severity of PTSD, anxiety, and depression were expressed as the IES-R, HADS anxiety, and HADS depression sum scores, respectively. Prevalence of probable PTSD, anxiety, and depression was defined as the proportion of patients scoring above the cut-off. Abbreviations: CI, confidence interval; COVID-19, coronavirus disease 2019; ICU, intensive care unit; OR, odds ratio; PTSD, post-traumatic stress disorder. Differences over time were calculated using mixed effects linear (for continuous outcomes) and logistic (for categorical outcomes) regression models, with time as independent variable. Differences between stratifications were analyzed using simple linear (for continuous outcomes) and logistic (for categorical outcomes) regression models.*“ (Results, Table 2, page 16, line 272)

And changed the legend of Table 3 accordingly:

“*Descriptive statistics of the HRQoL outcomes, stratified by COVID-19 diagnosis and ICU admission. Differences over time were calculated using mixed effects linear (for continuous outcomes) and logistic (for categorical outcomes) regression models, with time as independent variable. Abbreviations: CI, confidence interval; COVID-19, coronavirus disease 2019; EQ-5D, European quality of life 5 dimensions questionnaire; HRQoL, health-related quality of life; ICU, intensive care unit; MCS-36, mental component score of the RAND-36; OR, odds ratio; PCS-36, physical component score of the RAND-36; TTO, time trade-off; VAS, visual analogue scale. Differences between stratifications were analyzed using simple linear (for continuous outcomes) and logistic (for categorical outcomes) regression models.*” (Results, Table 3, page 18, line 299)

14.	Line 221. You have good reasons to refer to supp. materials because no significant associations were found. However you can inform the reader about this (similar at other places) in a more gentle way (for instance: “”Analyses showed that none of the assessed predictors were significantly associated with probable PTSD, probable anxiety, and probable depression (for the results we therefore refer to ……)”.

**R:** We agree and changed accordingly:

“*Table 4 and S9 depict the results of the exploration of predictors for the severity and prevalence of probable PTSD, anxiety and depression.* ” (Results, Predictors of psychological outcomes and quality of life, page 17, line 292-293)

“*None of the predictors were significantly associated with the prevalence of probable PTSD (Table S9).*” (Results, Predictors of psychological outcomes and quality of life, page 17, lines 297-298)

“*None of the predictors were significantly associated with the prevalence of probable anxiety (Table S9).*” (Results, Predictors of psychological outcomes and quality of life, page 19, line 304-305)

“*None of the predictors were significantly associated with the prevalence of probable depression (Table S9).*“ (Results, Predictors of psychological outcomes and quality of life, page 19, lines 311-312)

15.	Line 221. “natural decline” what do you mean with natural? It is a decline, and we do not know from your data that it is natural.

**R:** We agree and omitted natural throughout the manuscript.

16.	Table 1. Please insert zero’s (N and %) instead of blanks because blanks may suggest that a variable was not assessed among a subgroup.

**R:** We changed Table 1 accordingly.

17.	Table 2. The total numbers at T1 and T2 in this table do not match figure 1. Check or explain differences (or add info in figure).

**R:** We apologize for the untidiness and checked all numbers. The numbers now all match with those in Figure 1.

18.	In table 1 the ICU and nonICU numbers at baseline are 39 and 105. However in Table 2 at base line they are 32 and 216 (!). This is very confusing. Please correct and revise. (In Table 3 numbers are missing).

**R:** We apologize for the untidiness and corrected the number.

19.	For the analyses of the differences between the subgroups, you controlled for differences at baseline. Please clarify how many control variables were entered in each analyses (present also event-per-variable ratio) so readers can see that your analyses are justified (EVP higher than 10 as a rule of thumb).

**R:** We understand the concerns of the reviewer. In hindsight, we realize that we have indeed adjusted for too many characteristics. We therefore now first analyzed the association between at baseline differing characteristics using simple univariate linear (for continuous outcomes) or logistic (for categorical outcomes) regression models. In these, the outcome variable served as dependent variable, and the at baseline differing characteristics one-by-one as independent variables. All at baseline differing characteristics which showed a high association, i.e., a *p*-value ≤0.10, were used in simple multivariate linear (for continuous outcomes) or logistic (for categorical outcomes) regression models, to compare outcomes between stratifications, while adjusting for at baseline differing characteristics which could confound the outcome.

Using this method, the number of variables for which was controlled did not violate the event-per-variable ratio.

We described this in the statistical analysis section of the methods:

“*Differences in outcome measures between stratifications were analyzed using simple linear or logistic regression models, to adjust for at baseline differing characteristics, for continuous and categorical outcomes, respectively. We therefore first performed a simple univariate linear (for continuous outcomes) or logistic (for categorical outcomes) regression analyses, in which all at baseline differing characteristics were analyzed one-by-one. Variables that were associated with the outcome, i.e., a p-value ≤0.10, were added as independent variables to the simple multivariate regression models and were as such adjusted for.*” (Methods, statistical analysis, page 9, lines 195-200)

20.	Line 246. Work resumption. Again, this topic was hardly introduced in the intro (what dod we know from the literature about work resumption after hospitalization). Perhaps it is an idea to omit this topic from your manuscript.

**R:** We agree with the editor and omitted this topic from the manuscript.

21.	Table 4. This table suggest that IES-R and HADS scores at T1 were not predictive. Is this correct? (this is a very remarkable outcome).

**R:** The outcomes of the IES-R and HADS, both at 1 month (T1) and 3 months (T2) were both added as dependent variables, we therefore added time as independent variable. Table 4 depicts the outcomes of the mixed-effects linear regression model, in which the IES-R sum score, HADS anxiety score, and HADS depression score at both follow-up time-points served as dependent variables (using a longfile), while time and at baseline differing variables (see our answer to your previous comment) served as independent variables. As such, no conclusions can be drawn about a possible predictive nature of psychological outcomes at T1 from Table 4, as we did not examine this.

22.	Discussion. To help the reader please use more subheadings in your discussion section.

**R:** We agree with the editor and added the subheadings “Limitations” and “Conclusions” to our discussion section.

Although we fully agree with the editor that subheadings in a discussion may be of help to guide the reader through the discussion, in this discussion we feel that adding additional subheadings feels rather artificial and rather distracts than helps the reader.

23.	Line 268. See comment 1. I believe some rewriting is needed because of this study by Taquet et al. 2021.

**R:** We agree and added the following:

“*In a recent large retrospective study, COVID-19 was indeed associated with a higher risk of psychiatric outcomes as compared to influenza and respiratory tract infections (29). In our cohort however, psychological distress was largely similar between non-COVID-19 patients with respiratory symptoms and COVID-19 patients, and we did not observe COVID-19 positivity to be a predictor for severity of PTSD, anxiety, or depression. As such, our data collectively suggest that COVID-19 on its own has no major influence on psychological outcome. The contradictory findings of the Taquet and our study will largely be explained by the difference in study design (retrospective cohort vs. prospective observational cohort), the different outcome measures used (ICD-based diagnoses vs. questionnaire-based outcome) and comparing the covid-19 cohort with other control populations (influenza and respiratory tract infection vs. patient with symptoms suggestive of COVID-19 but COVID-19 PCR negatives).*” (Discussion, page 25, lines 369-378)

24.	Line 285. Ref. 41 is about life time prevalence (and retrospective!) and should not be used in this way. The prevalence do not seem very high compared to victims of f.i. accidents, medical errors, and (sexual) violence (see van der velden et al. 2020. [https://doi.org/10.1371/journal.pone.0232477](about:blank))

**R:** We apologize for the untidiness, and changed accordingly:

“*The observed prevalence of psychological distress in our cohort appears to be higher than in the general population during non-pandemic circumstances, and was similar to people suffering from PTSD-related symptoms after a traumatic event, such as theft, burglary, accidents and death of a significant other (41).*” (Discussion, page 24, lines 354-356)

25.	Line 331. “Baseline psychological status and HRQoL in our cohort was not available”. You mean before infection/hospitalization? (you used the word baseline to describe T1).

**R:** We agree and changed accordingly. This sentence now reads:

“*In our cohort, psychological status and HRQoL prior to hospitalization were not available and thus we cannot formally rule out that pre-existing psychological well-being was poorer, predisposing for impaired psychological recovery (47).*” (Discussion, page 25, lines 380-382)

“*First, the high incidence of psychological symptomatology and the poor HRQoL post-discharge may be either attributed to factors related to hospitalization or to baseline psychological imbalances, as psychological status and HRQoL of participants prior to hospitalization was not available.*” (Discussion, page 26, lines 407-410)

We however want to stress that T1 is not described as baseline, as T1 refers to the first follow-up time-point, which was one month after discharge from the hospital.

26.	Line 350-353. Should “Physicians should be aware of the psychological consequences of hospitalization during a pandemic but not only in those affected by COVID-19 or requiring ICU treatment” be “However, Physicians should be aware of the psychological consequences of hospitalization during a pandemic but not only in those affected by COVID-19 or requiring ICU treatment”.

**R:** We agree and changed accordingly.

27.	Upload all supplementary materials as separate files.

**R:** We have uploaded all supplementary materials as separate files as requested by the editor.

**Response to Reviewer #3:**

Second review: Psychological distress and health-related quality of life in patients after hospitalization during the COVID-19 pandemic: a single-center, observational study

I want to thank the authors for the good job done, especially on methodology. There is a significant improvement on the quality of the manuscript. However, some work need to be done to allow the paper to be published

Introduction

1. This section was reviewed partially. “This paper’s aim is to quantify psychological distress and to determine their HRQoL ”. The introduction needs a section to briefly discuss few different methods to assess mental health and HRQoL. This section will help non expert readers to understand and also give a good flavor to the reader

**R:** First, we thank the reviewer for his useful comments and suggestions.

With regard to the suggestion to briefly discuss few different methods to assess mental health and HRQoL, we believe that elaborating more on this matter will limit the focus and conciseness of the introduction, and does, in our opinion, not add value to the introduction.

We however understand that the choices made regarding the assessment of psychological distress and HRQoL remains a matter of debate, and therefore discussed this, although briefly in the discussion section:

“*Fourth, we assessed psychological well-being and HRQoL using self-report questionnaires. Although commonly used and extensively validated, formal assessment of psychologic disorders requires consultation with a psychologist or psychiatrist, and usage of self-reports may result in an overestimation of psychologic distress.*” (Discussion, page 26, lines 420-422)

Methodology

Authors have made significant progress in term of reporting methodology. However, data analysis part is not well reported;

it is not clear in the analysis which method used at each level, for example;

2. Line 172; “Differences in outcome measures between stratifications were analyzed using linear or logistic regression”. Clarify where you used linear and where you used logistic regression and clarify the logistic model used (i.e function or Equation). For logistic regression (binary outcomes), I was expecting to read the results with Odds ratios and confidence intervals. Please explain this!

**R:** First, we apologize for the unclarity. A similar comment was raised by the academic editor. To clarify; we used simple linear regression models to compare continuous outcomes between stratifications, and simple logistic regression models to compare dichotomous/categorical variables. Mixed effects linear regression models were used to analyze the course of psychological symptomatology, in means of the IES-R sum score, HADS anxiety score, and HADS depression score, and the course of HRQoL, in means of mental quality of life (MCS-36), physical quality of life (PCS-36), and overall HRQoL (EQ-5D TTO score), and we used mixed effect logistic regression models to analyze the course of the prevalence of probable PTSD, anxiety, and depression, as defined as patients scoring above the described cut-off value. Furthermore, for the identification of risk factors for psychological distress and HRQoL, we used mixed effects linear (for continuous outcomes) or logistic (for categorical outcomes) regression models.

We changed the description of this in the Statistical analysis section of the Methods:

“*Differences in outcome measures between stratifications were analyzed using simple linear or logistic regression models, to adjust for at baseline differing characteristics, for continuous and categorical outcomes, respectively. We therefore first performed a simple univariate linear (for continuous outcomes) or logistic (for categorical outcomes) regression analyses, in which all at baseline differing characteristics were analyzed one-by-one. Variables that were associated with the outcome, i.e., a p-value ≤0.10, were added as independent variables to the simple multivariate regression models and were as such adjusted for.*

*To identify possible predictors for the severity and prevalence of psychological distress and the overall, mental, and physical HRQoL, we first conducted univariate mixed effects regression analysis: a linear model for continuous outcomes and a logistic model for categorical outcomes. We used mixed effects regression models to adjust for intergroup (i.e., time) and intragroup differences (i.e., cohort and variables of interest). In these, the possible predictive variable served as independent variable one-by-one and the outcome of interest at both follow-up time points served as dependent variables. Secondly, all variables which showed a p-value ≤0.10 in the univariate mixed effects regression model were added to the multivariate mixed effects regression model to determine which variables significantly predicted the outcome.*” (Methods, Statistical analysis, page 9-10, lines 195-208)

Also, the reviewer rightfully points out that it was inappropriate to report the raw coefficients of the logistic regression models. We have changes these to OR’s and the corresponding confidence intervals:

“*We report the coefficient [95% CI], which implies the estimated mean difference, for linear models, and odds ratios (ORs), including its 95% CI, for logistic models.*” (Methods, Statistical analysis, page 10, lines 208-209)

3. Line 185-186 “Psychological outcomes of randomly selected non-responders were compared with those of responders using a linear or logistic regression model”. It is not clear where applied linear regression, where applied multiple and where applied logistic regression. Please clarify these by showing which statistical model used (i.e function or equation).

**R:** We apologize for the untidiness, and are convinced that we now better described how non-responders were analyzed:

“*Baseline characteristics of all non-responders were compared with those of responders using a Mann-Whitney U test for continuous variables and using a Fisher’s exact test for categorical variables. Psychological outcomes of randomly selected non-responders (who did not respond at both time-points) were compared with those of responders, and psychological outcomes of full responders were compared with those of partial responders (i.e., patients who only responded at one or three months), using simple linear or logistic regression models for continuous and categorical variables, respectively. In these, we adjusted for variables that were expected to confound the outcome and differed at baseline between stratifications as described above.*” (Methods, Statistical analysis, page 10, lines 210-216)

4. Please discuss with your analyst if there is a possibility of using “ Time to event outcomes regression” (cox proportional hazards model) and calculate hazard ratios for ICU versus non-ICU and COVID-19 versus non COVID-19 in the development or reduction of psychological distress from the time of discharge to one month time follow up!

**R:** We appreciate the suggestion of the reviewer and discussed this matter with our statistician (B. Boxma-de Klerk). Unfortunately, such an analysis requires us to know at what time point exactly someone started suffering from psychological distress, rather than if a patient reported psychological distress at a certain follow-up assessment. In our study, we only assessed psychological distress at two time-points: one month and three months after discharge. For patients who already reported psychological distress at one point, or for those who did not reported psychological distress at one month, but did at three months, we cannot know when during follow-up these symptoms first manifested. As such, it is not possible to use time to event outcomes regression.

Health related quality of life;

5. Clarify how you calculated utility scores and health states, then how did you do the crosswalk for the health states?

**R:** The time trade off (TTO) score of the EQ-5D is computed by giving a weight for eight possible answers for each item. These weights are subtracted from the maximum; which is 1. So, every patients starts with a EQ-5D TTO score of 1.00. For each answer given on one of the five domains, a certain weight is subtracted. For instance, for the mobility domains, no subtraction is conducted when a patients has no problems walking, 0.035 is subtracted when a patient has slight problems walking, 0.057 is subtracted when a patient has moderate problems with walking, 0.166 is subtracted when a patients has severe problems with walking, and 0.203 is subtracted when a patients is unable to walk. Every answer possibility has other weights, and an additional weight is subtracted when a patient reports the worse score on all five domains. These weights are country-specific, and published within the validation study of the EQ-5D in the Dutch population.

The computation of the EQ-5D utility/TTO score is described in the paper of Herdman *et al*. (2011) and Versteegh *et al*. (2016), to which we refer when we first mention the EQ-5D .

With regard to the MCS-36 and PCS-36; the calculations for these summary scales are extensively described in the paper of Ware *et al*. (1994). In summary; first all subscales are calculated, and subsequently these are normalized against the general population, so that the mean is 50 and the standard deviation is 10 in the general population. The mean of the subscales in its section represent the mental and physical component score.

As the EQ-5D and RAND-36 are the most commonly used instrument to determine a person’s quality of life, and the exact calculations of the health states/summary scales does not fall under the scope of the present study, we do not further elaborate on this within the manuscript.

6. Explain how you calculated the mean utility scores

**R:** We have explained the calculations behind the utility scores in the response to your previous comment.

7. Explain how you estimated QALYS lost due to psychological distress

**R:** We do not fully comprehend the reviewer’s comment, as we do not estimate and discuss any loss of QALYs due to psychological distress in the manuscript under review.

Results

All relevant or significant results (i. e significant P-values) in the tables in this section must be briefly commented below each table.

**R:** We indeed did not comment on all significant p-values within the manuscript to maintain conciseness.

Based on the comment of the reviewer, we added the following to the manuscript to address all significant outcomes of Table 1:

“*Table 1 depicts the most relevant baseline demographics and treatment-related characteristics; a full overview of all collected demographics and characteristics can be found in Table S8. The median age was 64 years (95% range 33-88), 106 (36%) were female, and a history of mental illness was reported by 27 of 201 patients (13%), who were willing to share this information. The median hospital length of stay (LOS) was 4 days (1-52) and 42 patients (14%) required ICU admission (median ICU LOS; 16 days [95% range 0-52]). Overall, 146 patients (50%) were SARS-CoV-2 positive. Non-COVID-19 patients were predominantly diagnosed with other pulmonary or cardiac illnesses (Table S8). COVID-19 patients were younger, had fewer comorbidities, more frequently worked prior to hospitalization, had a longer hospital and ICU LOS, were more frequently admitted to the ICU, had a higher SOFA score and lower P/F ratio on admission, more frequently received oxygen therapy and invasive ventilation for a longer duration, were more frequently mechanically ventilated in prone position and more frequently received a tracheotomy than non-COVID-19 patients (Table 1). Within COVID-19 patients, those who were admitted to the ICU had a longer hospital LOS, a higher SOFA score and lower P/F ratio, received oxygen therapy more frequently and more frequently received a tracheostomy (Table 1).*” (Results, Participants, page 12-14, lines 238-251).

We had already commented on all significant results from Table 2 and 3.

Based on the comment of the reviewer, we added the following to the manuscript to address all significant outcomes of Table 4 and 5:

“*Time after discharge (p=0.01), not being Caucasian (p<0.01) and having completed higher professional education were associated with the severity of PTSD in the univariate mixed effects regression analyses and were included in the multivariate mixed effects regression analysis. Of these,…*” (Results, Predictors of psychological distress and quality of life, page 17, lines 293-295)

“*Time after discharge (p=0.01), female gender (p=0.01), having completed higher professional education (p=0.01), employment status before hospitalization (p=0.08), and COVID-19 diagnosis (p=0.06) were associated with the severity of anxiety in the univariate mixed effects regression analyses and were included in the multivariate mixed effects regression analysis. Of these,…*“ (Results, Predictors of psychological distress and quality of life, page 19, lines 300-303)

“*Time after discharge (p=0.01), female gender (p=0.02), not being Caucasian (p=0.06), having completed higher professional (p<0.01) or university education (p=0.01), employment status before hospitalization (p=0.02), COVID-19 diagnosis (p=0.03) and ICU admission (p=0.07) were associated with the severity of depression in the univariate mixed effects regression analyses and were included in the multivariate mixed effects regression analysis. Of these,…*” (Results, Predictors of psychological distress and quality of life, page 19, lines 306-310)

“*Time after discharge (p=0.01), age (p<0.01), female gender (p<0.01), having completed higher professional (p<0.01) or university education (p<0.01) and COVID-19 diagnosis (p<0.01) were associated with overall HRQoL in the univariate mixed effects regression analyses and were included in the multivariate mixed effects regression analysis. Of these,…*” (Results, Predictors of psychological distress and quality of life, page 19, lines 313-316)

“*Time after discharge (p<0..01), female gender (p<0.01), being not Caucasian (p=0.05), having completed university education (p=0.03), being mechanically ventilated (p=0.03), ICU admission (p=0.01) and having more severe symptoms of PTSD (p<0.01), anxiety (p<0.01) and depression (p<0.01) were associated with mental HRQoL in the univariate mixed effects regression analyses and were included in the multivariate mixed effects regression analysis. Of these,…*” (Results, Predictors of psychological distress and quality of life, page 19, lines 321-325)

“*Lastly, time after discharge (p<0.01), age (p<0.01), female gender (p<0.01), employment status before hospitalization (p<0.01), hospital LOS (p=0.07), COVID-19 diagnosis (p<0.01) and having more severe symptoms of PTSD (p<0.01), anxiety (p<0.01) and depression (p<0.01) were associated with physical HRQoL in the univariate mixed effects regression analyses and were included in the multivariate mixed effects regression analysis. Of these,…* “ (Results, Predictors of psychological distress and quality of life, page 20, lines 328-331)

8. For example, “Table1: Baseline demographics and treatment-related characteristics of study population”. Where are comments of this table?

**R:** We have described all variables that were differing at baseline in the results section, as described in the answer to your previous comment.

9. Table2 “Psychological outcomes throughout follow-up” is confusing because it is not commented. I don’t see the relevance of this table. And if am not wrong, no significant results are reported in this table. And then, the bottom of this table reads logistic regression. With logistic regression results, one would expect to be reported Odds ratios (ORs) and confidence intervals. I suggest you remove this table because the results are confusing and are not explained therefore it does not add value unless if it is clearly commented.

**R:** We regret that the relevance of these tables was unclear, and a similar comment was raised by the academic editor. Table 2 and 3 depicts our primary and secondary outcome measures; Table 2 represents the severity and prevalence of psychological distress, as measured at both follow-up moments. The P-values provided regarding the comparison between stratifications are calculated using simple linear and logistic regression models, which is largely comparable with an ANCOVA, to adjust for at baseline differing variables, and the P-values regarding the change over time were calculated using mixed effects linear and logistic regression models.

To clarify this, we revised the legend of Table 2:

“*Descriptive statistics of the psychological distress outcomes, stratified by COVID-19 diagnosis and ICU admission. Severity of PTSD, anxiety, and depression were expressed as the IES-R, HADS anxiety, and HADS depression sum scores, respectively. Prevalence of probable PTSD, anxiety, and depression was defined as the proportion of patients scoring above the cut-off. Abbreviations: CI, confidence interval; COVID-19, coronavirus disease 2019; ICU, intensive care unit; OR, odds ratio; PTSD, post-traumatic stress disorder. Differences over time were calculated using mixed effects linear (for continuous outcomes) and logistic (for categorical outcomes) regression models, with time as independent variable. Differences between stratifications were analyzed using simple linear (for continuous outcomes) and logistic (for categorical outcomes) regression models.*“ (Results, Table 2, page 16, line 272)

And changed the legend of Table 3 accordingly:

“*Descriptive statistics of the HRQoL outcomes, stratified by COVID-19 diagnosis and ICU admission. Differences over time were calculated using mixed effects linear (for continuous outcomes) and logistic (for categorical outcomes) regression models, with time as independent variable. Abbreviations: CI, confidence interval; COVID-19, coronavirus disease 2019; EQ-5D, European quality of life 5 dimensions questionnaire; HRQoL, health-related quality of life; ICU, intensive care unit; MCS-36, mental component score of the RAND-36; OR, odds ratio; PCS-36, physical component score of the RAND-36; TTO, time trade-off; VAS, visual analogue scale. Differences between stratifications were analyzed using simple linear (for continuous outcomes) and logistic (for categorical outcomes) regression models.*” (Results, Table 3, page 18, line 299)

10. Table3, 4 and 5 are relevant but are not commented. Please comment important or significant results in these tables

**R:** We understand the confusion of the reviewer, as we indeed did not comment on the univariate analysis depicted in these tables. As described in your previous comment, we have now commented on all significant results in these tables.

Discussion

I am still not able to comment on discussion; results need to be worked on before reviewing the discussion part.

11. Intext citations and references don’t follow PLOS ONE guidelines. Please check on PLOS ONE referencing guidelines

**R:** We apologize for the untidiness, and fully copy-edited the references section so that all references follow the PLOS ONE guidelines.
